# Genetics and Nutrition Drive the Gut Microbiota Succession and Host-Transcriptome Interactions through the Gilthead Sea Bream (*Sparus aurata*) Production Cycle

**DOI:** 10.3390/biology11121744

**Published:** 2022-11-30

**Authors:** Fernando Naya-Català, M. Carla Piazzon, Silvia Torrecillas, Socorro Toxqui-Rodríguez, Josep À. Calduch-Giner, Ramón Fontanillas, Ariadna Sitjà-Bobadilla, Daniel Montero, Jaume Pérez-Sánchez

**Affiliations:** 1 Nutrigenomics and Fish Growth Endocrinology Group, Institute of Aquaculture Torre de la Sal (IATS, CSIC), 12595 Castellón, Spain; 2 Fish Pathology Group, Institute of Aquaculture Torre de la Sal (IATS, CSIC), 12595 Castellón, Spain; 3 Grupo de Investigación en Acuicultura (GIA), IU-ECOAQUA, Universidad de Las Palmas de Gran Canaria, Crta. Taliarte s/n, 35214 Telde, Las Palmas, Canary Islands, Spain; 4 Skretting Aquaculture Research Centre, 4016 Stavanger, Norway

**Keywords:** sea bream, selective breeding, nutrition, gut microbiota, host transcriptomics, intestinal motility, epithelial turnover, lipid metabolism, inflammatory response

## Abstract

**Simple Summary:**

The AquaIMPACT H2020 EU project aims at the integration of selective breeding and nutrition for improving the competitiveness of European Aquaculture, promoting at the same time the production of robust and high-quality fish with a limited environmental impact. The gut microbiota is one of criteria chosen to assess the achievement of such goals, and we evaluated herein how the gut microbiota changes with the diet along the production cycle in reference (REF) fish and fish genetically improved (GS) for growth. To better underscore the association of the host with its gut microbiota, we also deconvoluted the correlation of gut bacteria with the host intestinal gene expression profile, which highlighted the physiological processes that are independent or potentially related to changes in gut microbiota composition. The physiological relevance of these findings is discussed in relation to the different growth potentiality of the two fish populations analysed.

**Abstract:**

Fish genetically selected for growth (GS) and reference (REF) fish were fed with CTRL (15% FM, 5–7% FO) or FUTURE (7.5% FM, 10% poultry meal, 2.2% poultry oil + 2.5% DHA-algae oil) diets during a 12-months production cycle. Samples from initial (t_0_; November 2019), intermediate (t_1_; July 2020) and final (t_2_; November 2020) sampling points were used for Illumina 16S rRNA gene amplicon sequencing of the adherent microbiota of anterior intestine (AI). Samples from the same individuals (t_1_) were also used for the gene expression profiling of AI by RNA-seq, and subsequent correlation analyses with microbiota abundances. Discriminant analyses indicated the gut bacterial succession along the production cycle with the proliferation of some valuable taxa for facing seasonality and different developmental stages. An effect of genetic background was evidenced along time, decreasing through the progression of the trial, namely the gut microbiota of GS fish was less influenced by changes in diet composition. At the same time, these fish showed wider transcriptomic landmarks in the AI to cope with these changes. Our results highlighted an enhanced intestinal sphingolipid and phospholipid metabolism, epithelial turnover and intestinal motility in GS fish, which would favour their improved performance despite the lack of association with changes in gut microbiota composition. Furthermore, in GS fish, correlation analyses supported the involvement of different taxa with the down-regulated expression of pro-inflammatory markers and the boosting of markers of extracellular remodelling and response to bacterium. Altogether, these findings support the combined action of the gut microbiome and host transcriptionally mediated effects to preserve and improve gut health and function in a scenario of different growth performance and potentiality.

## 1. Introduction

The shortage of traditional marine feedstuffs promotes the use of new aquafeeds based on plant ingredients, insect proteins, animal-by-products, seaweeds and fermented products among other alternative and sustainable feed ingredients [1,2,3,4,5]. Meanwhile, the contribution of aquaculture production to the global aquatic food production volume is continuously increasing [6], and selective breeding is a widely applied practice to improve the performance and profitability of a wide range of farmed fish to meet the increasing demand of animal proteins for human food consumption [7,8]. Such improvements in fish breeding require novel tools and approaches, and new knowledge of genetic and nutrition interactions would contribute to the production of robust and healthy fish with an increased phenotypic plasticity when responding to environmental challenges [9,10,11,12]. In that sense, at the interplay between genetics and nutrition, the gut microbiota is now emerging as a reliable criterion to assess the attainment of selective breeding to improve the performance and competitiveness of farmed fish in a scenario of climate change [13].

Like in other animals, the gut microbiome of fish contains thousands of microbial species that establish a complex network of relationships among each other and with the host [14,15,16]. Indeed, both the transient and resident gut microbial communities produce a number of metabolites that impact the host functions not only at the local level, but also at a distant one [17,18]. As part of this complex puzzle, the modulatory effect of the diet on the intestinal microbiota has been extensively documented in some farmed fish, including gilthead sea bream (*Sparus aurata*) [2,19] and European sea bass (*Dicentrarchus labrax*) [20,21,22]. The same cannot be said for host genetics, whose influence on the composition and activity of gut microbiota remains less investigated. However, recent studies in gilthead sea bream pointed out that intensive selection for fast-growth co-selects for a plastic gut microbiota with a wider metabolic response to cope with changes in diet composition [23,24]. Links between gut microbiota and the host-transcriptome have also been established in gilthead sea bream, using targeted [17,18,22] or massive gene expression [25] approaches. However, most of this research is focused on stationary models in a given geographical location, and further research is needed to establish the temporal trends for most of the host-associated bacterial successions with changes in age, sex, season, nutrition and rearing conditions [26,27,28]. Certainly, the time-course of genetics and nutrition interactions have been addressed within the framework of the AquaIMPACT H2020 EU project, using genetically selected fish for accelerated growth that were fed through the production cycle with standard (commercial-based diet) or “future” (half reduction in fish meal, devoid of fish oil) diet formulations according to the principles of circular economy. Preliminary results from this AquaIMPACT feeding trial in the Canary Islands revealed that fish selected for growth not only displayed enhanced growth and feed conversion, but also demonstrated a better utilization of the alternative diet formulation in association with a higher fillet content of docosahexaenoic acid (DHA) [29]. Herein, using fish from the same feeding trial, we aimed to study the succession of the gilthead sea bream gut microbiota from juvenile to adult stages to delineate the main conjoint effects of genetic background and the diet composition on a temporal basis. Additionally, we focused on the association of the intestinal transcriptome and resident/adherent gut microbiota to elucidate the cooperative metabolic processes taking place in the gut.

## 2. Materials and Methods

### 2.1. Ethics Statement

All the experiments, fish manipulation and tissue samplings included in this manuscript were performed following the current Spanish (Royal Decree RD53/2013) and EU (2010/63/EU) laws. All the procedures described were also validated by the bioethical committee of the University of Las Palmas de Gran Canaria (OEBA_ULPGC_26/2019).

### 2.2. Diets

Two isoenergetic and isonitrogenous diets, with a varying granule size and composition, were formulated by Skretting Aquaculture Research Centre (Stavanger, Norway) according to the nutritional requirements for gilthead sea bream (Table 1). The control diet (CTRL) contained FM (15%) and terrestrial plants as the main protein sources. The alternative diet (FUTURE) was half-reduced in FM (7.5%) mostly due to the inclusion of poultry meal (10%). FO was added at 5.7–7.6% in the CTRL diet, whereas the FUTURE diet was completely devoid of FO. FO was instead replaced by poultry oil and DHA-algae oil. The two diets were formulated to have the same content (8.5%) in fatty acid methyl esters (FAME) corresponding to the sum of eicosapentaenoic acid (EPA) and DHA amounts in the diets.

### 2.3. Broodstock Crosses

A population of 6122 adult fish from the Canary Islands at the third generation of the National Breeding Program (PROGENSA^®^) were evaluated for growth. The estimated breeding values (EBV) ranged between −159.14 for reference fish (REF) and +223.18 for the genetically improved fish (GS) with an average value of 8.59 and a standard deviation value of 52.84. A subset of 196 fish (96 fish per broodstock) was then selected for breeding with values for the EBV varying from −25.95 in the group of REF fish to +39.68 in the group of GS fish, comprising almost the 47% of the evaluated population.

### 2.4. Experimental Fish, Husbandry Conditions and Sampling

The progeny from either REF fish or GS fish were kept under similar conditions during the pre-weaning, weaning and early juvenile growing phases (15–20 g). Fish were then fed in 1000 L tanks (20 tanks, 50 fish per tank, 250 fish per group) with CTRL or FUTURE diets for 12 months in the experimental facilities of ECOAQUA (Taliarte, Las Palmas de Gran Canaria, Spain) under natural photoperiod and temperature conditions at this latitude (27°59′ N; 15°22′ W). During the feeding period (November 2019–November 2020), fish were manually fed until apparent satiation 4 times a day, 6 days per week, and the amount of feed was registered daily. The amount of estimated feed ingested per fish was not significantly different among treatments, ranging between 0.68 and 0.85 g feed/100 g body weight/day. The initial (t_0_; November 2019; average 10 g body weight, BW), intermediate (t_1_; July 2020; average 200 g BW) and final (t_2_; November 2020; average 350 g BW) sampling points were established. At each sampling time, fish were sampled following overnight fasting, anaesthetized with clove oil anaesthesia (0.02 mL/L; Guinama S.L; Spain, Ref. Mg83168) and euthanized using an overdose of natural clove oil. At t_0_, 18 fish from REF and GS fish were retrieved (7–11 per group). At t_1_ and t_2_ sampling points, 9–10 fish from each diet and genetic condition were taken. In total, 94 fish were randomly sampled, and a tissue portion of anterior intestine (AI), immediately after the pyloric caeca, was rapidly excised, fixed in RNA later and stored at −80 °C until RNA extraction. The rest of the AI was opened and washed with sterile phosphate-buffered saline (PBS) to remove non-adherent materials and bacteria. This tissue portion was transferred to a clean Petri dish, and the internal mucus was scraped out with the blunt end of a sterile scalpel. DNA extraction was performed immediately after sampling. The anterior portion of the intestine was chosen due to its double role in both the gut microbiota and host activity. Autochthonous mucosal adherent bacteria have an impact on the metabolism and absorption of nutrients [30]. Additionally, the anterior portion of the intestine is known to develop a higher digestive enzyme activity [31], which can help to obtain the correlation between the gut microbiota and the host transcriptional activity, which is one of the main objectives of this work.

### 2.5. DNA and RNA Extraction

DNA extraction for microbiota analysis (all sampling times) was performed using the High Pure PCR Template Preparation Kit (Sigma-Aldrich, St. Louis, MO, United States), including a lysozyme lysis step. Samples from t_1_ were also processed for gene expression analysis, and total RNA from AI was extracted with the MagMAXTM-96 Total RNA Isolation Kit (Applied Biosystems, Foster City, CA, USA). The RNA concentration and purity were determined using a Nanodrop 2000c tool (Thermo Scientific, Wilmington, DE, USA). The quality and integrity of the isolated RNA were verified with an Agilent Bioanalyzer 2100 total RNA Nano series II chip (Agilent, Amstelveen, The Netherlands), yielding RNA integrity numbers (RINs) of between 8 and 10. Extracted DNA and RNA were stored at −80 °C until sequencing.

### 2.6. 16S rRNA Sequencing and Bioinformatic Analysis

In order to characterize the gut-adherent microbiota, amplification and sequencing of the V3–4 hypervariable region of the 16S rRNA gene were performed using the Illumina MiSeq platform (Illumina, San Diego, CA, USA) in a 2 × 300 paired-end (PE) design. The procedure was carried out at the Genomics Unit from the Madrid Science Park Foundation (FPCM), and details of PCR amplification and amplicon sequencing can be found elsewhere [26]. The bioinformatics approach involved a quality assessment with FastQC and Prinseq (http://www.bioinformatics.babraham.ac.uk/projects/fastqc/, accessed on 3 November 2021), and bacterial taxonomy assignment using VSEARCH and BLAST [32,33] against the Ribosomal Database Project (RDP) release 18 [34], accepting as optimal the alignments with strong similarity (≥90%) and query coverage (≥90%). Inferred metagenome analyses were performed using Picrust [35].

All 36 RNA-seq libraries were sequenced on the Illumina Hiseq 2500 platform with a 2 × 150 nucleotides paired-end (PE) read format according to the manufacturer’s protocol at the GENEWIZ company (Leiden, Germany). A quality analysis was performed with FastQC, and libraries were filtered with Trimmomatic [36] for quality >28 and <5% of Ns in the sequence. Then, libraries were mapped and annotated with STAR [37], using the CSIC gilthead sea bream draft genome as the reference library [38]. Unique transcripts hit counts were calculated by using featureCounts from the Subread package [39]. All the sequencing data obtained along this work can be accessed at Sequence Read Archive (SRA), using the Bioproject accession number PRJNA876784 (BioSample DNA accession numbers: SAMN30672395-488; Biosample RNA accession numbers: SAMN30672489-524).

### 2.7. Statistics

Rarefaction curves, coverage ratio (ratio between observed and Chao1 indexes values), species richness estimates, and alpha diversity indexes of microbial communities were obtained using the R package phyloseq [40]. Differences in richness, diversity indexes, and phylum and core microbiota abundance were determined using the Kruskal–Wallis test (Dunn’s post-test, *p* < 0.05). Beta diversity across groups was tested with PERMANOVA (*adonis*, 10,000 random permutations, *p* < 0.05). The sequential application of a supervised partial-least square discriminant analysis (PLS-DA) using EZinfo v3.0 (Umetrics, Umeå, Sweden) and a hierarchical clustering (*hclust* R function) was used to show the separation of the experimental groups. All the markers (gene or OTUs) introduced in the PLS-DA models were ordered according to their variable importance in the projection (VIP) values, and selecting the markers with a VIP value ≥ 1 as the main discriminant variables, since they achieved the complete clustering of the conditions [41,42,43]. Outliers in EZinfo v3.0 were reported at a Hotelling’s T2 statistic threshold of 99%. Validation of the models was performed with a validation test of 500 random permutations, performed using the Bioconductor R package *ropls* [44], using a *p* < 0.05 threshold for the R2Y (fit of the model) and Q2 (prediction of the model) parameters. Linear discriminant analysis (LDA) effect size (LEfSe) was used to identify significant differences in metagenomic taxa between the experimental sampling times [45] using the online tool Galaxy v1.2 [46]. OTUs with VIP ≥ 1 were included in this analysis, and statistically significant differences were retrieved with the factorial Kruskal–Wallis test (Wilcoxon post-test; α = 0.05). Significant inferred metagenomics pathways were considered using an FDR-corrected significance threshold of 0.05.

For RNA-seq analyses, differentially expressed (DE) transcripts were retrieved using DESeq2 [47] with an FDR-corrected significance threshold of 0.05. For individual correlation analyses with microbiota data (t_1_), Spearman correlation coefficients were calculated for DE transcripts and OTUs with VIP ≥ 1, as reported elsewhere [48], and considered significant at a *p* < 0.01. All the networks included in this work were built and visualized with Cytoscape v3.8.2 [49].

Fisher test-based over-representation analyses of BP-GO and KEGG terms were implemented in the goseq R package [50] and statistical significance was accepted at FDR < 0.05. The hierarchy of over-represented GO-BP terms was retrieved using GOATools [51] and GO-BP were clustered according to their common ancestor in Gene Ontology. The relationships between enriched GO-BP and KEGG terms according to their shared transcripts were performed using the *runGSA* function of the piano R package [52], and the resulting networks were visualized with Cytoscape v3.8.2 [49]. Functional protein–protein association networks were retrieved with The Search Tool for the Retrieval of Interacting Genes (STRING) database [53], being considered statistically significant at FDR of < 0.05 and with a confidence score of > 0.7, using zebrafish (*Danio rerio*) as the reference species.

## 3. Results

### 3.1. The Gut Microbiome of the Gilthead Sea Bream along Its Production Cycle

**Figure 1:**
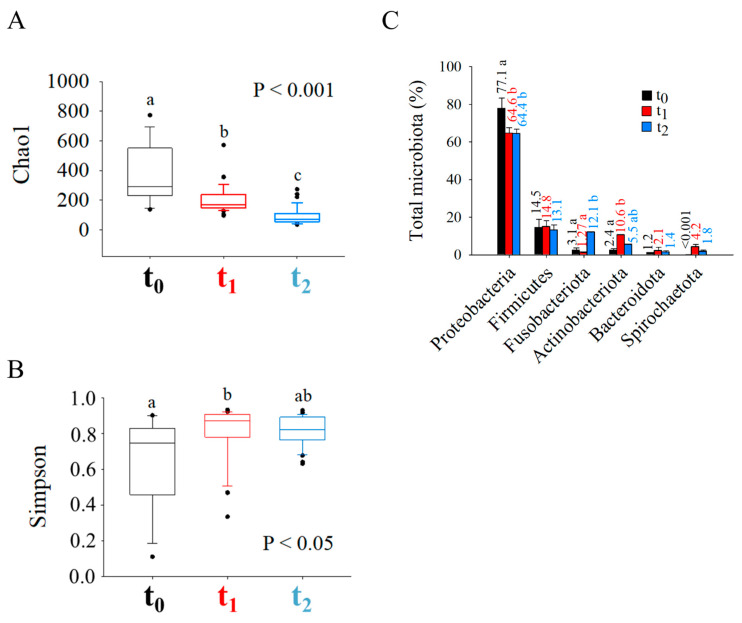
Box plots representing the median (min-max) of richness estimate [(**A**) Chao1] and diversity index [(**B**) Simpson] of the gut microbial populations of gilthead sea bream found at each sampling point: t_0_ (n = 18), t_1_ (n = 36), and t_2_ (n = 40). Different letters indicate significant differences among the groups (Kruskal–Wallis + Dunn’s post-test, *p* < 0.05). (**C**) Bar chart representing the relative abundance of bacterial phyla at each one of the experimental sampling points (t_0_, t_1_, and t_2_). Only the phyla that are present in at least 1% in one of the groups are represented. Numbers above the bars indicate the mean percentage of abundance. Different letters indicate significant differences among the groups (Kruskal–Wallis + Dunn’s post-test, *p* < 0.05).

Illumina sequencing and taxonomic assignment of ~10 M high-quality 16S rRNA reads from the 94 analysed fish (Supplementary Table S1; Supplementary Figure S1) facilitated the identification of 1892 OTUs at 97% identity, with a mean observed/expected OTUs ratio of 70%. Taking into account the entire population, regardless of the diet and host genetic background, the Chao1 richness index (Figure 1A) decreased progressively along time, whereas the opposite occurred with the α-diversity Simpson index (Figure 1B), which significantly increased at t_1_ and t_2_ sampling points in comparison to the beginning of the trial. At the phylum level (Figure 1C), Proteobacteria was the most abundant (64–78%) bacteria category in all sampling points, though a significant decrease at t_1_ and t_2_ was detected in comparison to t_0_. This drop in Proteobacteria was balanced with a significant increase in Actinobacteriota at t_1_ (~10.6%) and of Fusobacteriota at t_2_ (~12%). Firmicutes (13–15%), Bacteroidota (1.2–2.1%), and Spirochaetota (2–4%) were also found in the analysis, but remained stable along the trial.

A total of 228 OTUs were found in at least one individual of each one of the three sampling points, constituting the core microbiota of gilthead sea bream along the production cycle (Supplementary Figure S2A). Specifically, 25 OTUs (>1% in at least one of the three sampling times) represented at least the 35% of the overall microbiota in each sampling (Supplementary Figure S2B,C). At t_0_, taxa assigned to the *Sphingomonas* genus were the most abundant within the core microbes, whereas at the intermediate sampling (t_1_), the decrease in Proteobacteria fueled an increase in the Actinobacteriota *Propionibacterium*, *Corynebacterium*, and *Arthrobacter*, of the Spirochaetota *Brevinema* and of the Caulobacteraceae family. At the end of the production cycle (t_2_), the increase in Fusobacteriota was mainly due to the increase in the *Cetobacterium* core community. Of note, *Photobacterium*, *Aquabacterium*, and *Staphylococcus* genera increased through host development, whereas the *Reyranella* genus remained constant with a significant proportion of ~8% during the entire trial.

**Figure 2:**
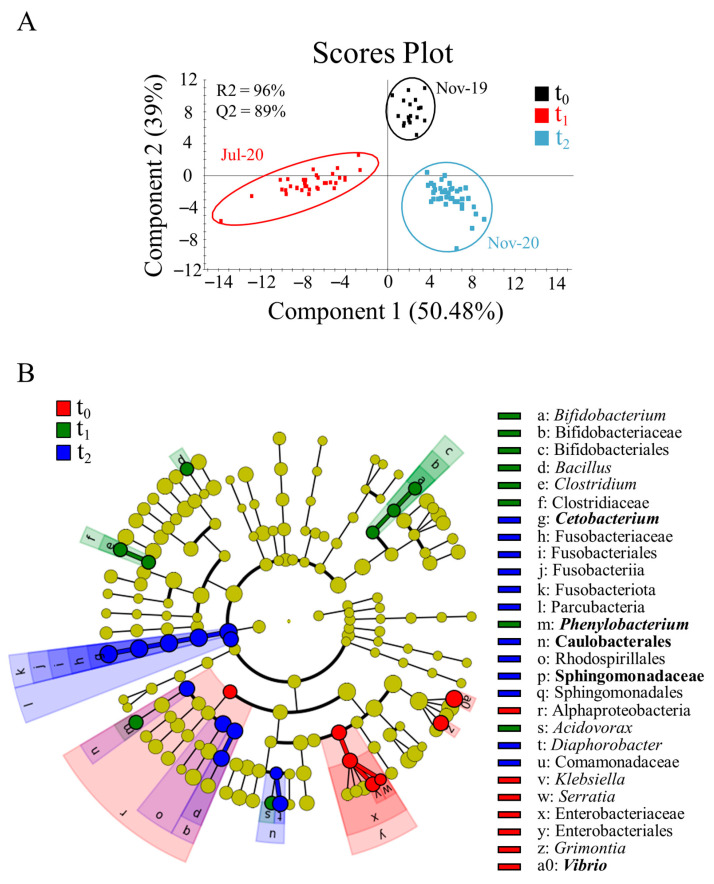
(**A**) Two-dimensional partial least-square discriminant analysis (PLS-DA) scores plot showing the differentiation of gut microbiota of gilthead sea bream over the production cycle regardless of diet. The validation permutational test can be found in Supplementary Figure S3A. (**B**) Cladogram for the linear discriminant analysis effect size (LEfSe) with the OTUs (VIP ≥ 1) that most likely drive differences in t_0_ (red), t_1_ (green), and t_2_ (blue). Results show the OTU classification at the level of phylum, class, order, family, genus and species from the inside to the outside. Taxon in bold indicates taxa belonging to the core microbiota.

Statistical differences (F = 3.9692, R^2^ = 0.1169, *p* = 0.001) were detected in the bacterial beta-diversity through time; to study these differences in more detail, we used a validated PLS-DA to render the separation of the three experimental sampling points (Figure 2A; Supplementary Figure S3A). The first component of this model (50.5% explained variance) separated fish from t_1_ from those of t_0_ and t_2_, depicting a putative seasonal effect (summer vs. autumn) over gut microbial composition. The second component (39% explained variance) separated the three groups, describing the changing gut microbiota with the increase in fish size through development. Among the total 150 OTUs with VIP ≥ 1 driving the separation of the groups (Supplementary Table S2A), the LEfSe analysis revealed a predominance of OTUs assigned to the Alphaproteobacteria class, Enterobacteriaceae family, and *Klebsiella*, *Serratia*, *Grimontia* and *Vibrio* genera (Figure 2B) at t_0_. The genera *Bacillus*, *Clostridium*, *Phenylobacterium* and *Acidivorax* were associated with t_1_. The increased abundance of the Sphingomonadales and Rhodospirillales orders, and *Diaphorobacter* and *Cetobacterium* genera was significant at t_2_.

The inferred metagenome analysis displayed a total of 110 OTUs (VIP ≥ 1) whose genomes could be potentially associated with the expression of transcripts involved in the differentially represented pathways (FDR < 0.05). A considerable difference in pathways was found between t_1_ and t_0_ (117), t_2_ and t_0_ (73), and t_2_ and t_1_ (132). For more information, the complete list of differentially expressed pathways can be accessed in Supplementary Table S3.

### 3.2. Genetic- and Nutritional-Mediated Effects on Gut Microbiota

**Figure 3:**
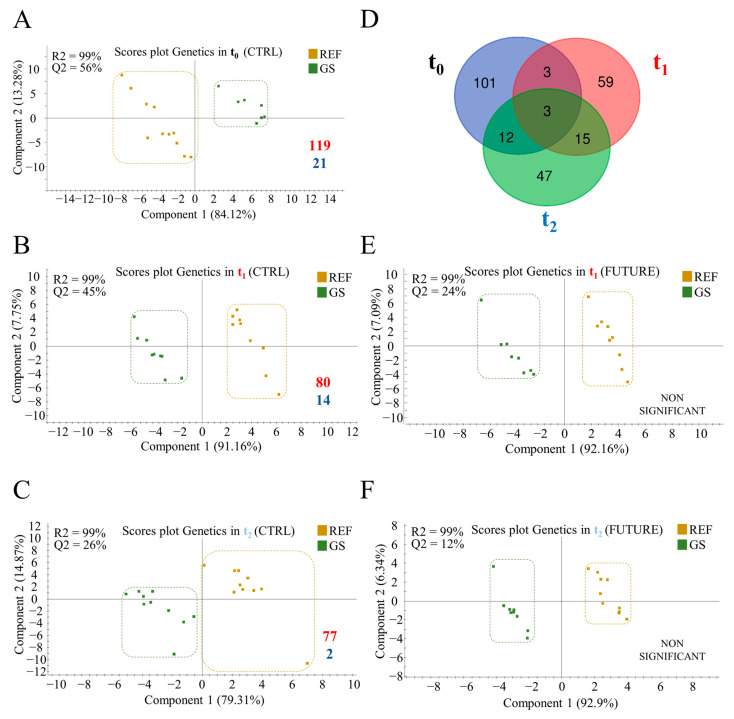
Two-dimensional partial least-square discriminant analysis (PLS-DA) scores plot assessing the effect of genetic background on gut microbiota of gilthead sea bream at the beginning of trial (**A**), fish fed the CTRL diet at t_1_ (**B**), and t_2_ (**C**), and fish fed the FUTURE diet in t_1_ (**E**), and t_2_ (**F**). The validation permutation test for (**A**–**C**) models can be found in Supplementary Figure S3B–D, respectively. NON SIGNIFICANT indicates non-validated models by permutation test. (**D**) Venn diagram showing unique and shared operational taxonomic units (OTUs) driving the separation of the respective groups in (**A**–**C**). Numbers in the bottom right of the plots indicate the number of discriminant OTUs (VIP ≥ 1) (red) driving the separation between groups, and the number of inferred pathways (blue) from each set of discriminant OTUs.

The genetic-mediated effects on gut microbiota were clearly evidenced at t_0_, where discriminant analyses separated GS from REF fish, showing how genetics can shape the microbiome of this species. The degree to which PLS-DA was able to mark this separation remained constant along time, but progressively decreased at t_1_ and t_2_ sampling points in fish fed the CTRL diet (Figure 3A–C; Supplementary Figure S3B–D). The number of discriminant and separating OTUs also suffered this decline, from 119 at t_0_, to 80 at t_1_, and 77 at t_2_, with these taxa being specific in this sampling time; only *Sphingomonas*, *Reyranella* and *Clostridium* were detected in all the three sampling times (Supplementary Table S2B–D; Figure 3D). As in the number of discriminant OTUs, a decrease was also found in the number of inferred pathways when comparing the GS and REF groups, from 21 at t_0_, to 14 at t_1_, and only 2 at t_2_ (Supplementary Table S4). The same trend was found with the FUTURE diet, but the multivariate analysis did to significantly discriminate the microbiota of GS and REF fish at any sampling time (Figure 3E,F).

**Figure 4:**
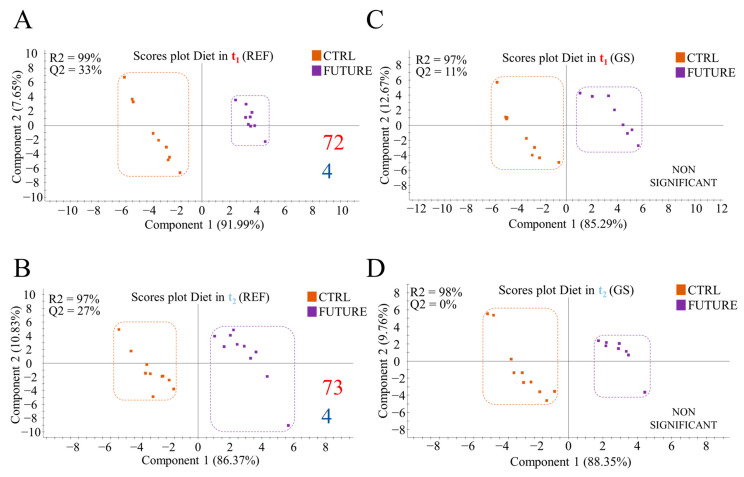
Two-dimensional partial least-square discriminant analysis (PLS-DA) scores plot assessing the effect of nutritional background in gilthead sea bream fed the CTRL diet at t_1_ (**A**) and t_2_ (**B**), and fish fed the FUTURE diet in t_1_ (**C**), and t_2_ (**D**). The validation permutation test for (**A**–**C**) models can be found in Supplementary Figure S3E,F respectively. NON SIGNIFICANT indicates non-validated models by permutation test. Numbers in the bottom right of the plots indicate the number of discriminant OTUs (VIP ≥ 1) (red), and the number of inferred pathways (blue) driving the separation between groups.

Conversely, the nutritionally mediated effects of the FUTURE diet on gut microbiota were amplified in REF fish. Certainly, this diet induced a consistent shift in the gut microbiota composition in REF fish (Figure 4A,B; Supplementary Figure S3E,F), but not in the GS fish (Figure 4C,D), which were able to maintain their gut microbiome structure regardless of diet composition. Across time (t_1_, t_2_), the number of discriminant OTUs (72–73) for GS and REF genotypes in fish fed the FUTURE diet remained stable (Supplementary Table S2E,F). The discriminant and highly abundant (VIP ≥ 1, >1% in at least one of the groups) *Photobacterium*, *Reyranella*, *Streptococcus* and *Brevinema* genera drove the separation of the two dietary groups at both sampling times (Supplementary Figure S4A). However, most OTUs with discriminant value were specific for each sampling point; the pathway-analysis for the inferred metagenome highlighted a clear divergence of the four over-represented pathways at each sampling time (Supplementary Figure S4B).

### 3.3. Analysis of RNA-seq Libraries by Stringent FDR and Correlation Analyses

**Figure 5:**
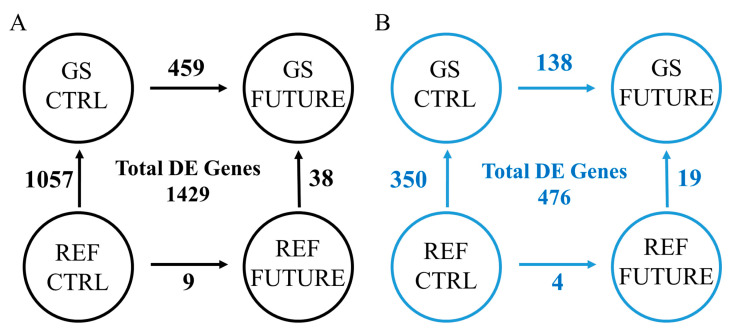
Differentially expressed genes at t_1_ in the anterior intestine of gilthead sea bream with a different genetic (GS, REF) and nutritional background (CTRL, FUTURE). (**A**) Number of differentially expressed transcripts (FDR < 0.05) for each comparison among groups. (**B**) Number of differentially expressed transcripts correlating with changes in gut microbiota composition (*p* < 0.01).

Given the above findings in gut microbiota composition, the intermediate sampling time was chosen as the most informative sampling point to disclose the microbiota and host-transcriptome interactions. Approximately 1000 million PE reads were obtained from the 36 sequenced samples using RNA-seq, with an average of ~28 million reads per sample. After trimming and quality filtering, around 1% of all reads were discarded, with the remaining reads ranging between 21 million (6.3 Gb) and 34 million (10.2 Gb) among the experimental groups (Supplementary Table S5). Up to 93% of these pre-processed reads were mapped against the reference genome, and unique hits counts were associated with 41,582 intestinal transcripts, corresponding to 18,522 unique descriptions (UD). The differential expression analysis discerned 1429 transcripts (1218 UD) significantly changing among comparisons. Among them, 1057 (913 UD) and 459 (415 UD) transcripts were differentially regulated between GS-CTRL and REF-CTRL fish, and between GS-FUTURE and GS-CTRL fish, respectively (Figure 5A). Only 38 DE transcripts (38 UD) marked the difference between GS-FUTURE and REF-FUTURE fish, further decreasing to 9 DE transcripts (9 UD) when comparing REF-FUTURE and REF-CTRL fish (for more details, see Supplementary Table S6). As a validation procedure, six genes covering a wide range of up- and down-regulation between GS-FUTURE and GS-CTRL were selected, and their fold-change values calculated using real-time PCR were quite consistent (*r* = 0.92) with those of the RNA-seq analysis (Supplementary Table S7). 

A total of 1018 significant associations (*p* < 0.01) were established between 96 OTUs and 476 DE transcripts (449 UD) (Supplementary Table S8; Figure 5B). The remaining 953 DE transcripts did not have any significant association with gut microbiota. These two lists of DE transcripts were studied independently in further enrichment and protein–protein interaction analyses.

### 3.4. Intestinal Over-Represented Processes

**Figure 6:**
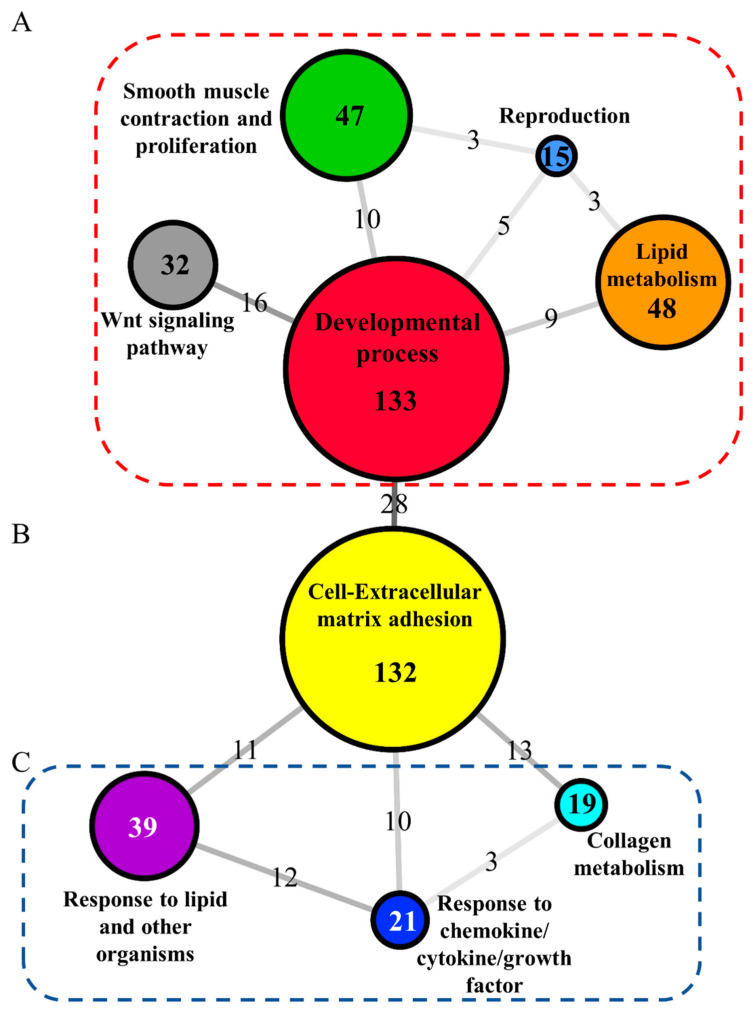
Network layout representing the associations between the supra-categories of over-represented GO-BP and KEGG terms of gilthead sea bream genes according to their shared allocated genes. (**A**) Network with the supra-categories associated to differentially expressed genes without correlation with microbiota (red box). (**B**) Network with the supra-categories associated to differentially expressed genes with and without correlation with microbiota. (**C**) Network with the supra-categories associated to differentially expressed genes with correlation with microbiota (blue box). Node colours show the representative name of the supra-category. Numbers in the center of the nodes represents the number of genes allocated to each supra-category. Numbers over the edges and shading represent the number of shared genes between two supra-categories.

The over-representation analyses of the microbiota-independent list of transcripts (953) displayed 33 and 19 GO-BP and KEGG unique terms, respectively (Supplementary Table S6). A total of 269 DE transcripts were present in several over-represented categories, which were clustered in 6 supra-categories (Figure 6A), including developmental process (133 transcripts allocated to 8 GO-BP over-represented terms), lipid metabolism (48 transcripts to 9 GO-BP), smooth muscle contraction and proliferation (47 transcripts to 3 GO-BP), the Wnt signalling pathway (32 transcripts to 3 GO-BP), and reproduction (15 transcripts to 1 GO-BP). The supra-category cell-extracellular matrix adhesion (132 transcripts to 6 GO-BP) included genes dependent as well as independent of gut microbiota correlations (Figure 6B). The over-representation analysis of the microbiota-correlated DE transcripts (476) discerned 15 and 3 GO-BP and KEGG unique terms, respectively (Supplementary Table S6). A total of 116 DE transcripts were present in several over-represented categories, which were clustered into 4 supra-categories (Figure 6C), including response to lipid and other organisms (39 transcripts allocated to 5 GO-BP over-represented terms), response to chemokine/cytokine/growth factor (21 transcripts to 4 GO-BP), and collagen metabolism (19 transcripts to 3 GO-BP).

### 3.5. Protein-Protein Network Analysis with Intestinal Transcripts Not Correlated with Microbiota

**Figure 7:**
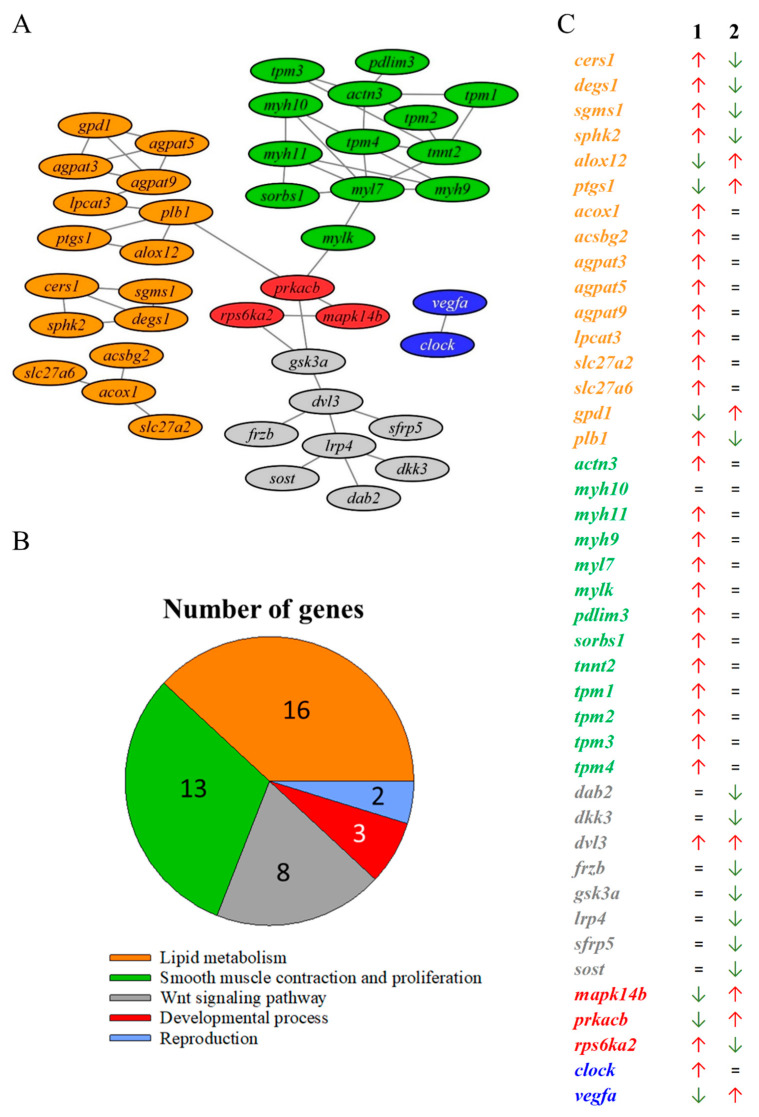
(**A**) Protein-protein interaction plot between gilthead sea bream genes involved in enriched processes. Edges between nodes show significant relations (FDR < 0.05; STRING confidence score > 0.7). (**B**) Pie chart showing the number of abundant OTUs related to each one of this three supra-categories. (**C**) Expression patterns of genes connected by the STRING plot. Symbols represent the gene expression log_2_FC values direction in the respective comparison stated in the letters above the columns. Green arrows indicate down-regulation, red arrows indicate up-regulation and “=” indicates no variation. Colours represent genes related to the following enriched biological process supra-category of Figure 6: Lipid metabolism (orange), Reproduction (blue), smooth muscle contraction and proliferation (green), Developmental process (red), and Wnt signalling pathway (grey). Numbers above columns indicate the comparison in the RNA-seq analysis (1: GS-CTRL vs. REF-CTRL; 2: GS-FUTURE vs. GS-CTRL). The comparisons GS-FUTURE vs. REF-FUTURE and REF-FUTURE vs. REF-CTRL were not included because all the genes in the figure remained unaltered in them.

A total of 53 significant protein interactions were disclosed within the list of DE transcripts without microbiota correlations (Figure 7A). The nodes in the network were reduced to sixteen UD involved in lipid metabolism supra-category, thirteen UD in smooth muscle contraction and proliferation, eight in Wnt signalling pathway, three UD in developmental process, and two UD in reproduction (Figure 7B). Within each supra-category, the allocated transcripts showed a significant alteration of the expression in the comparisons GS-CTRL vs. REF-CTRL, and GS-FUTURE vs. REF-FUTURE (Figure 7C). Transcripts related to the sphingolipid metabolism (*cers1*, *degs1*, *sgms1* and *sphk2*) were found to be up-regulated in GS-CTRL fish in comparison to REF-CTRL, but down-regulated in GS-FUTURE in comparison to GS-CTRL. In comparison to REF-CTRL, GS-CTRL fish showed the up-regulated expression of markers of β-oxidation of very long chain fatty acids (VLCFA) (*acox1*, *acsbg2*, *slc27a2* and *slc27a6*), and phospholipid transmethylation (*agpat3*, *agpat5*, *agpat9* and *lpcat3*). The same expression pattern was found in twelve out of the thirteen transcripts of the smooth muscle contraction supra-category (*actn3*, *myh9*, *myh10*, *myh11*, *myl7*, *mylk*, *pdlim3*, *sorbs1*, *tpm1*, *tpm2*, *tpm3* and *tpm4*). All these markers did not suffer any expression change in GS with the changing of diet. Molecules involved in the synthesis of arachidonic acid (*alox12* and *ptgs1*) were down-regulated in GS fish fed the CTRL diet in comparison to GS fed FUTURE diet, and REF fish fed the CTRL diet. The core transcript of the Wnt signalling pathway (*dvl3*) was up-regulated in selected fish and several of its antagonists (*lrp4*, *dab2*, *gsk3a*, *sfrp5*, *dkk3*, *frzb* and *sost*) shared a down-regulation in GS fish fed FUTURE diet in comparison to the same lineage fish fed the CTRL diet. These genes did not experience any significant change when comparisons were made between GS and REF fish fed the CTRL diet.

### 3.6. Linking Gut Microbial Abundances and Host Transcriptomic Patterns

**Figure 8:**
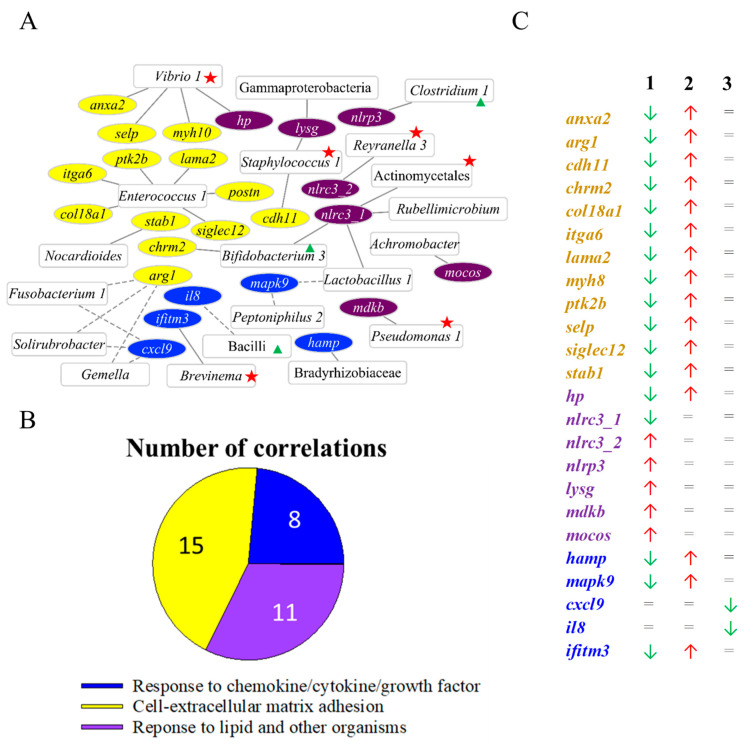
(**A**) Correlation network showing significant positive (straight lines) and negative (dotted lines) correlations (Spearman, *p* < 0.01) between discriminant (VIP ≥ 1) and abundant (≥1% in at least one of the groups) OTUs (squares), and gilthead sea bream DE transcripts belonging to the enriched supra-categories Response to chemokine/cytokine/growth factor (blue ellipses), Cell-extracellular matrix adhesion (yellow ellipses), and Response to lipid and other organisms (purple ellipses). Taxa belonging to core microbiota (red stars) and appearing in the LefSE analysis (green triangles) were highlighted. (**B**) Pie chart showing the number of abundant OTUs related to each one of this three supra-categories. (**C**) Expression patterns of genes plotted in the network. Green arrows indicate down-regulation, red arrows indicate up-regulation and “=” indicates no variation. Colours represent genes related to the following enriched biological process supra-category of Figure 6: Cell-extracellular matrix adhesion (yellow), Response to chemokine/cytokine/growth factor (dark blue), and Response to lipid and other organisms (purple). Numbers above columns indicate the comparison in the RNA-seq analysis (1: GS-CTRL vs. REF-CTRL; 2: GS-FUTURE vs. GS-CTRL; 3: GS-FUTURE vs. REF-FUTURE). The comparison REF-FUTURE vs. REF-CTRL was not included because all the genes in the figure remained unaltered in it.

A total of 34 out of 1018 significant correlations were established between 20 abundant bacteria (>1% in at least one of the groups REF-CTRL, GS-CTRL and GS-FUTURE) and 25 intestinal DE transcripts disclosed in enriched supra-categories (Figure 8A). This procedure displayed fifteen correlations between seven abundant OTUs and thirteen DE transcripts (13 UD) allocated to the cell-extracellular matrix adhesion supra-category. Response to chemokine/cytokine/growth factor and response to lipid and other organism supra-categories were also present, with eight and eleven correlations between eight and eleven abundant OTUs and five and seven DE transcripts, respectively (Figure 8B). At the same time, five correlated OTUs (*Vibrio* 1, *Reyranella* 3, *Brevinema*, *Pseudomonas* 1, and *Staphylococcus* 1) were identified as part of the core microbiota at the genus-level (Supplementary Figure S2). *Bifidobacterium* 3 and *Clostridium* 1 genera were highly abundant in fish sampled at t_1_, as a specific feature of this intermediate sampling time (Figure 2B).

Within each supra-category, the allocated transcripts showed a significant alteration of the expression in the comparisons GS-CTRL vs. REF-CTRL, GS-FUTURE vs. REF-FUTURE, and GS-FUTURE vs. REF-FUTURE (Figure 8C). The transcripts of the main components of the extracellular matrix (*anxa2*, *arg1*, *cdh11*, *chrm2*, *col18a1*, *itga6*, *lama2*, *myh8*, *ptk2b*, *selp*, *siglec12*, *stab1*) were down-regulated in GS fish fed the CTRL diet in comparison to GS fed the FUTURE diet, and REF fish fed the CTRL diet. These DE transcripts also showed positive correlations with *Enterococcus* 1, *Vibrio* 1, *Nocardioides*, *Staphylococcus* 1 and *Bifidobacterium* 3 genera. The same expression pattern was found in the growth factor responsive genes *mapk8*, *hamp* and *ifitm3*, which correlated positively with *Brevinema* and Bradhyrhizobiaceae, and negatively with *Lactobacillus* 1 and *Peptoniphilus* 2. The pro-inflammatory markers *il8* and *cxcl9* were down-regulated in GS fish fed the FUTURE diet in comparison to the REF-FUTURE group, showing negative associations with Bacilli-related OTUs (Bacilli and *Gemella*), *Solirubrobacter* and *Fusobacterium* 1. Bacterium-responsive genes (*mdkb*, *lysg*, *mocos*) were up-regulated in the GS-CTRL group in comparison to REF-CTRL fish, being positively correlated with Gammaproteobacteria, *Staphylococcus* 1, *Pseudomonas* 1 and *Achromobacter*. The same pattern was found for the inflammasome activator *nlrp3*, positively correlated with *Clostridium* 1. On the contrary, the monocyte-derived inflammation alleviators (*hp*, *nlrc3*) were down-regulated in GS-CTRL fish in comparison to the rest of the groups, showing a positive correlation with Actinomycetales and *Reyranella* 3.

## 4. Discussion

The host-associated bacteria succession during early life and first feeding, season and sex reversal has been already established in the protandrous fish gilthead sea bream [26,28,54]. In agreement with previous studies in gibel carp (*Carassius auratus gibelio*) [55] and Atlantic salmon (*Salmo salar*) [56], the changing pattern in gut microbiota composition was also found herein through the production cycle of gilthead sea bream (Figure 1 and Figure 2, Supplementary Figure S2). This temporal succession was already evident after the phylum-level taxonomic assignment (Figure 1C), which showed how the decrease in Proteobacteria, the usual dominant phylum in the farmed fish gut [57], gave way to an increase in Actinobacteriota at t_1_ and Fusobacteriota at t_2_, two phyla related with the host gut and liver health [58,59]. Further analyses (Figure 2; Supplementary Figure S2) also showed that the dominance of Proteobacteria at t_0_ was mainly due to OTUs assigned to *Vibrio* (22.6%) and *Sphingomonas* (4.5%) genera. These taxa are usually abundant in the gut of marine fish [60,61] and gilthead sea bream in particular [62], but their role is controversial as they correspond to wide and diverse taxonomic groups that can be implicated in both positive or pathogenic functions [63,64,65]. Otherwise, up to five predominant taxa (>1% in at least one of the sampling points) drove the most prominent changes of gut microbiota at the intermediate sampling point: Caulobacteraceae (mainly *Phenylobacterium*) (7.6%), *Brevinema* (4.1%), and the Actinobacteriota *Propionibacterium* (2.7%), *Corynebacterium* (1.1%) and *Arthrobacter* (1.1%). Interestingly, all these genera were identified as transient core taxa in Nile tilapia and zebrafish studies [66,67], highly influenced by the habitat and the seasonality of wild life in Nile tilapia and tench (*Tinca tinca*) [68,69]. Lastly, at the end of the trial, the most dramatic change in gut microbiota composition was the increase in Fusobacteriota (12.1%), mainly due to the *Cetobacterium* genus (11.5%). This taxon increased through development in Southern catfish (*Silurus meridionalis*) [70] and zebrafish [71]. Altogether, these results confirm and extend the notion of a dynamic microbiome colonization of the gut of almost all the studied animals, in which the decreasing amount of the most abundant phyla was concomitant with the proliferation of some valuable taxa for facing seasonality and different developmental stages, according to the host necessities.

Recent studies in model fish species [72,73] and farmed fish [74,75] highlighted the influence of host genetics upon the composition of gut microbiome. In the present study, this observation becomes evident from the beginning of the trial (Figure 3), and it was especially persistent in fish fed the CTRL diet with a high discriminant value of some abundant taxa (>1%) such as *Sphingomonas*, *Reyranella*, and *Clostridium*, which also shared a higher proportion in the GS fish. The difference in the genus *Sphingomonas* was more evident at the beginning of the trial (∆4%) than at t_1_ and t_2_ (∆1% and ∆0.5%, respectively), suggesting that the pathogenic opportunism of *Sphingomonas* [76] and its correlation with pro-inflammatory genes [17] can be diluted as host life advances in this fish species. The opposite trend was found for *Reyranella*, which increased minimally in GS fish at t_0_ (∆0.6%), and more intensely at t_1_ and t_2_ (∆8%). This bacteria lineage is associated with a positive impact on host survival [77], possibly due to its ability to maintain gut homeostasis by reducing the quantity of nitrates [78], which can partly explain the stability of this core taxa (7.5–8.5%) along the entire trial in this study. Otherwise, as seen before for PROGENSA^®^ selected fish [23], the genus *Clostridium* was also increased in the GS fish (∆6% along the trial). The Clostridiales order is a known promoter of the production of short-chain fatty acids (SCFAs), related to anti-inflammatory properties and disease resistance [18,79]. Therefore, it is tempting to consider the abundance of this taxa as a suitable indicator of the impact of gilthead sea bream selection programs on gut health, regardless of seasonality and developmental stage.

The interaction between diet and genetics remains relatively poorly studied in fish [23,75,80]. However, it is generally accepted that the high replacement of dietary marine feedstuffs (FM/FO) has very often had an impact on growth performance [81,82,83,84] and intestinal health [23,85] due, at least in part, to changes in gut microbiota [22,24]. Moreover, there is now evidence that the offspring of F1/F2 crosses for fast growing families in the PROGENSA^®^ selection program disclosed a more resilient and plastic microbiota in fish grow out in the Mediterranean aquaculture infrastructure of IATS-CSIC [23]. With the increase in the pressure of genetic selection, the achieved results with the F4 PROGENSA^®^ offspring in a Canary Islands trial helps us to reinforce this idea. Indeed, in the present study, the dietary intervention only induced a clear shift in the gut microbial composition of REF fish (Figure 4A,B), whereas the diet effect was masked in GS fish (Figure 4C,D). This finding probably indicates that GS fish do not need to change the composition of their gut microbiota to a high extent to modify their microbial activity to cope with changes in diet composition. It must be noted that the microbial composition of feeds and their potential influence in gut microbiome [86] was not determined; however, our present results are in accordance with metatranscriptomic analyses that highlighted that the metabolic plasticity of microbiota is higher for the PROGENSA^®^ offspring in fish families selected for fast growth [24]. Moreover, this functional feature involved changes in both bacteria and fungi transcripts, which highly enlarges the catalogue of microbial functions in the fish intestine. In the present study, this functional plasticity was also highlighted on a temporal basis, as evidenced the observation that the inferred function for changing bacteria with diet in REF fish changed over time at intermediate and final sampling times (Supplementary Figure S4A). In fact, only 11 out of 134 OTUs changed at both sampling times, as exemplified for the highly abundant *Photobacterium, Reyranella, Brevinema* and *Streptococcus*. In previous studies, these genera were triggered by low FM/FO diets in both gilthead sea bream and rainbow trout [2,87]. For gilthead sea bream in particular, the increase in *Streptococcus* and *Brevinema* can be linked to the use of DHA-algae oil as a main FO replacer [17], which suggests some association of gut bacteria with the source of dietary oil.

Microarray and RNA-seq methodologies were widely applied to detect changes in the intestinal transcriptome of fish in an unbiased and non-targeted manner [88,89,90]. Herein, we intersected the intestinal gene expression patterns with the abundance of gut microbes, putting together the matching samples from t_1_, the sampling time with more noticeable microbial alterations by genetics and nutrition interactions. Like gut microbiota, the AI transcriptome of GS and REF fish was highly regulated by genetics and diet, with 1057 transcripts varying between GS and REF fish fed the CTRL diet, though only 38 transcripts had a discriminant role in fish with a different genetic background when fed the FUTURE diet (Figure 5A). Similarly, the nutritionally regulated genes decreased from 459 DE transcripts (415 UD) in GS to 9 DE transcripts (9 UD) in REF fish when comparisons were made for a given lineage between CTRL- and FUTURE-fed fish. This host transcriptional regulation is, thereby, similar to that reported for the genetically and nutritionally regulated composition of gut microbiota in this and previous metagenomics [23] and metatranscriptomic [24] studies after selective breeding for fast-growth in the PROGENSA^®^ program. Therefore, it appears conclusive that both the gut microbiota and host-transcriptomic patterns are structurally stable, but at the same time more plastic is present at the functional level in fish with an improved growth potentiality.

At a closer look, the protein interaction plot with the transcripts not correlated with gut microbiota indicates lipid metabolism, smooth muscle contraction, and the Wnt signaling pathway as the main differentially regulated processes in our genetics and nutrition interaction model (Figure 7A). Relatedly, sphingolipids in the digestive system are responsible of numerous important physiological and pathological processes, providing structural integrity to gut endothelial cells, regulating nutrient absorption, and acting as receptors for many microbial antigens and their toxins [91,92]. Herein, up to four genes implicated in the biosynthesis of sphingolipids (*cers1*, *degs1*, *sgms1*, *sphk2*) interacted in the network and were up-regulated in GS-CTRL fish (Figure 7B). Previous serum untargeted metabolomics studies pointed out the impact of plant-based diets over the sphingolipid metabolism [93], and the results of the present study also suggest a genetic and nutrition interaction effect in this process. Phospholipids are also major constituents of cell membranes, and their remodeling has a major impact upon the digestive system [94]. The Land’s cycle, mainly orchestrated by the acyltransferase protein family, regulates this remodeling through the hydrolysis and acetylation of phospholipids [95,96,97]. Here, up to four acyltransferases involved in this cycle were interacting in the protein plot, being up-regulated in GS fish. Probably, the most interesting feature of this gene category is the increased expression of *lpcat3*, which would facilitate the intestinal fatty acid and cholesterol absorption, evidenced by its required expression for the survival on a high-fat diet in a rodent model [96]. How these nutritionally mediated changes in the intestinal gene expression pattern of GS fish can contribute to mediate, at least in part, their enhanced fillet retention of DHA [29] remains to be fully understood. In any case, the results of growth performance indicated an improved feed conversion in GS fish [29], as it was also reported for fish growth across the production cycle in the Mediterranean region, with continuous growth rather than explosive growth spurts in summer [83]. It is important to consider that changes in the transcriptomic response can only be associated with the different fish genetics or to the entire diet formulation. To generate more insights into the role of specific nutrients (e.g., poultry meal and oil, DHA-algae oil), a meta-analysis with novel computational models based on the Bayesian networks approach is being undertaken. Gastrointestinal physiology is also adapted to the different growth features, with GS fish showing an enhanced enzyme-digestive activity (unpublished results), as part of the different adaptive features to improve and/or accelerate the digestive process, including enhanced intestinal motility and intestinal epithelial cell turnover [98,99,100]. At the transcriptomic level, the increase in gut motility would be shared in the present study by the up-regulated expression in GS fish of transcripts related with smooth muscle contraction, with a key role in propelling and mixing the bolus of food [101]. The link between gut epithelial turnover and improved growth performance was driven herein by the activation in GS fish of Wnt signaling, closely related with epithelium renewal, and tissue repair and homeostasis [102,103]. Indeed, regardless of the dietary treatment, GS fish boosted the expression of one of the main activators of this route, the *dvl3* [104]. This gene was further up-regulated in GS-FUTURE fish in association with the repression of several antagonists of the Wnt pathway (*dab2*, *gsk3a*, *sfrp5*, *dkk3*, *frzb*, *sost*) [105,106], which reinforced the role of this pathway in the gut epithelial restoration of fish fed diet formulations with drawback inflammatory effects in gilthead sea bream [85]. Herein, this was supported by the up-regulation in GS-FUTURE fish of pro-inflammatory arachidonic acid markers (*alox12*, *ptgs1*) [107,108].

Other changes in the intestinal GS fish transcriptome were related to gut microbial oscillations (Figure 8A,B). This was pointed out by the up-regulation in this group of fish of several molecular markers of response to bacterium, represented in this study by *lysg*, *mdkb* and *mocos* [109,110,111]. Such genes showed a strong positive correlation with *Staphylococcus* 1, *Pseudomonas* 1 and *Achromobacter,* which may act as the main targets/activator of this host defense system. GS-CTRL also potentiated the control of the inflammatory processes derived from intestinal lipopolysaccharides (LPS) [112]. The over-accumulation of bacterial pro-inflammatory cytosolic LPS [113] causes the development of sepsis in animals and fish [114,115] through the activation of the NLRP3 inflammasome, the main mechanism of cytokine production [116]. This process can be reverted by SCFAs [117], and their increased production by some more abundant taxa (*Clostridium*) in GS fish fed the CTRL diet would counteract the enhanced expression of the NLRP3 inflammasome-core gene *nlrp3*. The taxa *Reyranella*, *Rubellimicrobium*, *Lactobacillus* and *Bifidobacterium* also emerge as GS-related taxa in fish fed with the FUTURE diet, due to their positive correlations with the *nlrc3* gene, a recognized negative regulator of the LPS-derived inflammatory pathways [118]. In fact, some of these taxa have been recently reviewed [119] as mediators of host gut immunity through the formation of butyric acid and exopolysaccharides in the case of Firmicutes [120] and *Bifidobacterium* or *Lactobacillus* [121], respectively. We also observed a decrease in the expression of the pro-inflammatory *il8* and *cxcl9* genes in GS-FUTURE fish in comparison to the REF-FUTURE group, concurrent with the increase in Bacilli-related taxa (Bacilli and *Gemella*). Previous studies in gilthead sea bream using qPCR-arrays have already stated this association [17,18], and the current analysis reinforces the anti-inflammatory and anti-oxidant properties of this taxonomic class [122], commonly used as a probiotic to improve the growth and gut health of livestock fish [123,124]. Lastly, a total of 10 transcripts of the principal protein components of the ECM (*anxa2*, *chrm2*, *col18a1*, *lama2*, *itga6*, *myh10*, *postn*, *ptk2b*, *selp* and *stab1*) [125] were up-regulated in GS-FUTURE fish in comparison to GS-CTRL, suggesting an enhanced remodeling of the extracellular architecture. The extensive positive correlation of *Vibrio 1* and *Enterococcus 1* with most of these genes would support the involvement of microbiota in this regulatory process, as previously reported for these same bacterial taxa in models of crab [126] and humans [127].

## 5. Conclusions

**Figure 9:**
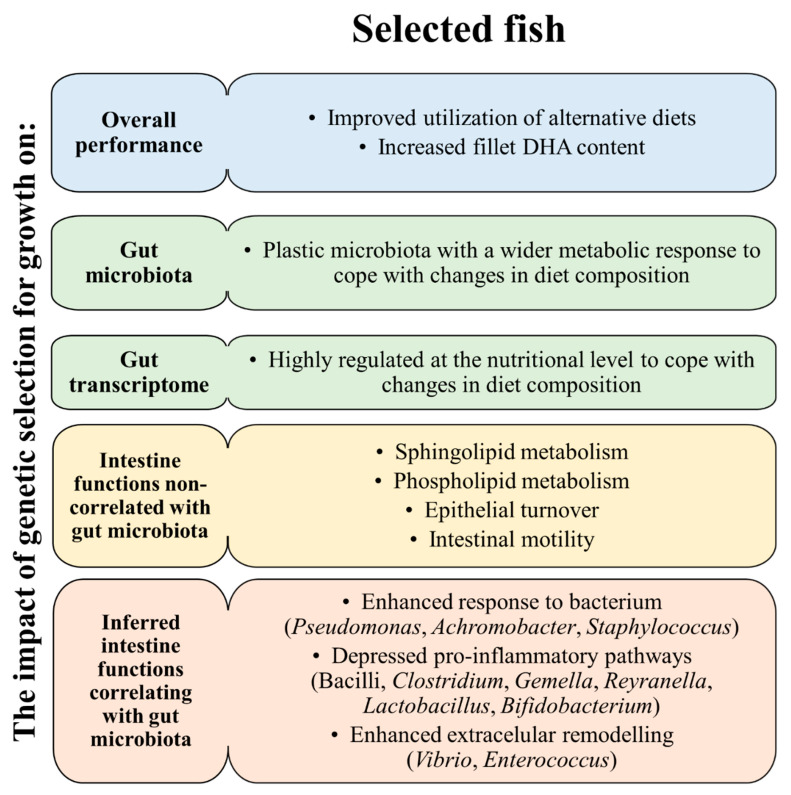
Schematic representation of integrative responses attained in AQUAIMPACT H2020 EU project for the genetically improved gilthead sea bream within the PROGENSA^®^ selective program for overall performance, gut microbiota and intestinal transcriptome patterns with/without correlations with the microbial community.

The results of this study highlighted the improved microbiota plasticity of GS fish within the PROGENSA^®^ breeding program, confirming and extending the suitability of gut microbiota studies to unravel some metabolic and functional advantages of genetically improved fish to better adapt to environmental changes (see Figure 9). This was assessed on a temporal basis contributing to establishing the core microbiota of gilthead sea beam across the production cycle, and how it is associated to nutritionally- and/or genetically-mediated changes in host transcriptomics. Among the most remarkable transcriptomic outcomes, the lipid metabolism and Wnt signaling appeared to fuel the gut epithelium regeneration with an increased intestinal motility, which would facilitate the digestive process of GS fish. Additionally, host–microbiome correlations suggested the increased responsive nature of GS fish to bacteria-derived complications in the intestine, as well as the repression of pro-inflammatory markers, in accordance with the proliferation of gut health-related taxa such as Bacilli, *Clostridium*, *Gemella*, *Reyranella*, *Lactobacillus*, and *Bifidobacterium*. In a practical sense, this work reinforces the role of gut microbiota to unveil the impact of genetic selection programs and to disclose specific trends and taxa that could help in the management of selective breeding in fish. At the same time, the combination of data from different omics platforms helped us to integrate the information between the “two genomes” of gilthead sea bream, its DNA sequence and its gut microbiome, in order to provide new insights into the cooperative processes occurring in the gut between these two biological systems.

## Figures and Tables

**Table 1:** Ingredients and chemical composition of experimental diets for gilthead sea bream feeding with varying pellet sizes (1.8, 4 and 6 mm).

Ingredients (%)	CTRL			FUTURE		
	1.8	4	6	1.8	4	6
Fish meal	15	15	15	7.50	7.50	7.50
Poultry meal				10	10	10
Hi Pro Soy bean meal	6.50	6	6	9.29	5.08	6
Soy protein concentrate	20	17	19	20	17	12
Faba bean dehulled	8	8	8	8	8	8
Corn gluten	7.95	5	5	4	5	5
Wheat gluten	12.10	14.90	8.55	18.34	14.44	12.87
Wheat	12.1	19.11	21.35	11.14	19.09	22.67
Fish oil	5.75	6.71	7.67			
Poultry oil				2.21	2.1	2.44
DHA-rich Algae oil				2.22	2.53	2.85
Rapeseed oil	3.79	5.16	6.50	4.65	6.52	7.86
Vitamin and mineral preMIX	0.30	0.30	0.30	0.30	0.30	0.30
Phosphate	0.75	0.82	0.63	0.35	0.44	0.51
Lecithin	2	2	2	2	2	2
*Chemical composition*						
Crude Protein (%)	48.29	43.02	39.56	48.64	43.51	40.20
Crude Lipid (%)	16	18	20	16	18	20
EPA + DHA (% FAME)	8.2	8.2	8.2	8.5	8.5	8.5

## Data Availability

Raw sequencing data are available at NCBI’s Sequence Read Archive under accession PRJNA876784 (BioSample accession numbers: SAMN30672395-524).

## Supplementary Materials

The following supporting information can be downloaded at: https://www.mdpi.com/article/10.3390/biology11121744/s1, Supplementary Figure S1. Rarefaction curves obtained from the sequencing data of the 94 samples included in this study; Supplementary Figure S2. Core microbiota. (A) Venn diagram depicting unique and shared OTUs among different age groups. (B) Venn diagram depicting unique and shared OTUs among different age groups including only OTUs with a relative abundance of >1% in, at least, one of the groups. (C) Abundance table representing the core 25 OTUs from (B). The presence within each group, in percentage, is expressed and the mean abundance of each OTU within each group, in percentage, is highlighted with a color scale being red the highest and green the lowest. The “-” indicates a proportion below 0.001%. Different letters indicate significant differences among the groups (Kruskal–Wallis + Dunn’s post-test, *p* < 0.05). The last row represents the percentage of the total microbiota per group constituted by the core microbiota. t_0_, t_1_, and t_2_ correspond to November 2019, July 2020, and November 2020 sampling points, respectively; Supplementary Figure S3. Validation (permutation test, 500 permutations) of the PLS-DA models shown in this study; Supplementary Figure S4. (A) Venn diagram showing unique and shared operational taxonomic units (OTUs) driving the separation of the respective groups in Figure 4A,B. (B) Results from the pathway analysis performed with the predicted metagenome obtained from the discriminant OTUs with VIP ≥ 1. Only differential pathways (padj < 0.05) are shown in the figure. Supplementary Table S1. Table showing the detailed 16S amplicon-based sequencing data obtained in this study, as well as the number of observed OTUs and the species richness estimator (Chao1), obtained for each sample; Supplementary Table S2. List of discriminant OTUs (VIP ≥ 1) of the PLS-DA models presented along the study and its corresponding VIP values; Supplementary Table S3. Pathway analysis results from predicted metagenomes. Values represent the log2 fold change of the comparisons between the fish from each one of the experimental times (A) t_1_ vs. t_0_, (B) t_2_ vs. t_0_, and (C) t_2_ vs. t_1_; Supplementary Table S4. Pathway analysis results from predicted metagenomes. Values represent the log2 fold change of the comparisons between GS and REF fish at t_0_ and fish fed CTRL diet at t_1_, and t_2_; Supplementary Table S5. Table showing the detailed RNA-sequencing data obtained in this study, as well as the percentage of unique and multiple mapping rates; Supplementary Table S6. List of transcripts DE in, at least, one comparison made between experimental groups. Information of gene annotation, log_2_FC data and GO-BP and KEGG enrichment is included; Supplementary Table S7. Comparison between RNA-seq results and real-time PCR validation. Supplementary Table S8. List of significant (*p* < 0.01) correlations between the anterior intestine OTUs and DE genes obtained at t_1_.

## References

[1] Hua K., Cobcroft J.M., Cole A., Condon K., Jerry D.R., Mangott A., Praeger C., Vucko M.J., Zeng C., Zenger K. (2019). The Future of Aquatic Protein: Implications for Protein Sources in Aquaculture Diets. One Earth.

[2] Naya-Català F., Wiggers G.A., Piazzon M.C., López-Martínez M.I., Estensoro I., Calduch-Giner J.A., Martínez-Cuesta M.C., Requena T., Sitjà-Bobadilla A., Miguel M. (2021). Modulation of Gilthead Sea Bream Gut Microbiota by a Bioactive Egg White Hydrolysate: Interactions Between Bacteria and Host Lipid Metabolism. Front. Mar. Sci..

[3] Solé-Jiménez P., Naya-Català F., Piazzon M.C., Estensoro I., Calduch-Giner J., Sitjà-Bobadilla A., Van Mullem D., Pérez-Sánchez J. (2021). Reshaping of Gut Microbiota in Gilthead Sea Bream Fed Microbial and Processed Animal Proteins as the Main Dietary Protein Source. Front. Mar. Sci..

[4] Carvalho M., Montero D., Rosenlund G., Fontanillas R., Ginés R., Izquierdo M. (2020). Effective Complete Replacement of Fish Oil by Combining Poultry and Microalgae Oils in Practical Diets for Gilthead Sea Bream (*Sparus aurata*) Fingerlings. Aquaculture.

[5] Porcino N., Genovese L. (2022). Review on Alternative Meals for Gilthead Seabream, *Sparus aurata*. Aquac. Res..

[6] Henriksson P.J.G., Troell M., Banks L.K., Belton B., Beveridge M.C.M., Klinger D.H., Pelletier N., Phillips M.J., Tran N. (2021). Interventions for Improving the Productivity and Environmental Performance of Global Aquaculture for Future Food Security. One Earth.

[7] Boudry P., Allal F., Aslam M.L., Bargelloni L., Bean T.P., Brard-Fudulea S., Brieuc M.S.O., Calboli F.C.F., Gilbey J., Haffray P. (2021). Current Status and Potential of Genomic Selection to Improve Selective Breeding in the Main Aquaculture Species of International Council for the Exploration of the Sea (ICES) Member Countries. Aquac. Reports.

[8] Gjedrem T., Robinson N., Rye M. (2012). The Importance of Selective Breeding in Aquaculture to Meet Future Demands for Animal Protein: A Review. Aquaculture.

[9] Jennings S., Stentiford G.D., Leocadio A.M., Jeffery K.R., Metcalfe J.D., Katsiadaki I., Auchterlonie N.A., Mangi S.C., Pinnegar J.K., Ellis T. (2016). Aquatic Food Security: Insights into Challenges and Solutions from an Analysis of Interactions between Fisheries, Aquaculture, Food Safety, Human Health, Fish and Human Welfare, Economy and Environment. Fish Fish..

[10] Kause A., Kiessling A., Martin S.A.M., Houlihan D., Ruohonen K. (2016). Genetic Improvement of Feed Conversion Ratio via Indirect Selection against Lipid Deposition in Farmed Rainbow Trout (*Oncorhynchus Mykiss Walbaum*). Br. J. Nutr..

[11] Ahmed N., Thompson S., Glaser M. (2019). Global Aquaculture Productivity, Environmental Sustainability, and Climate Change Adaptability. Environ. Manag..

[12] Vandeputte M., Gagnaire P.A., Allal F. (2019). The European Sea Bass: A Key Marine Fish Model in the Wild and in Aquaculture. Anim. Genet..

[13] Terova G., Naya-Català F., Rimoldi S., Piazzon M.C., Torrecillas S., Toxqui M.S., Fontanillas R., Calduch-Giner J., Hostins B., Sitjà-Bobadilla A. (2022). Highlights From Gut Microbiota Survey in Farmed Fish-European Sea Bass and Gilthead Sea Bream Case Studies. Aquac. Eur..

[14] Merrifield D.L., Rodiles A. (2015). The Fish Microbiome and Its Interactions with Mucosal Tissues.

[15] Lin W., Djukovic A., Mathur D., Xavier J.B. (2021). Listening in on the Conversation between the Human Gut Microbiome and Its Host. Curr. Opin. Microbiol..

[16] Schroeder B.O., Bäckhed F. (2016). Signals from the Gut Microbiota to Distant Organs in Physiology and Disease. Nat. Med..

[17] Naya-Català F., do Vale Pereira G., Piazzon M.C., Fernandes A.M., Calduch-Giner J.A., Sitjà-Bobadilla A., Conceição L.E.C., Pérez-Sánchez J. (2021). Cross-Talk Between Intestinal Microbiota and Host Gene Expression in Gilthead Sea Bream (*Sparus aurata*) Juveniles: Insights in Fish Feeds for Increased Circularity and Resource Utilization. Front. Physiol..

[18] Piazzon M.C., Naya-Català F., Pereira G.V., Estensoro I., Del Pozo R., Calduch-Giner J.A., Nuez-Ortín W.G., Palenzuela O., Sitjà-Bobadilla A., Dias J. (2022). A Novel Fish Meal-Free Diet Formulation Supports Proper Growth and Does Not Impair Intestinal Parasite Susceptibility in Gilthead Sea Bream (*Sparus aurata*) with a Reshape of Gut Microbiota and Tissue-Specific Gene Expression Patterns. Aquaculture.

[19] Piazzon M.C., Calduch-Giner J.A., Fouz B., Estensoro I., Simó-Mirabet P., Puyalto M., Karalazos V., Palenzuela O., Sitjà-Bobadilla A., Pérez-Sánchez J. (2017). Under Control: How a Dietary Additive Can Restore the Gut Microbiome and Proteomic Profile, and Improve Disease Resilience in a Marine Teleostean Fish Fed Vegetable Diets. Microbiome.

[20] Torrecillas S., Mompel D., Caballero M.J., Montero D., Merrifield D., Rodiles A., Robaina L., Zamorano M.J., Karalazos V., Kaushik S. (2017). Effect of Fishmeal and Fish Oil Replacement by Vegetable Meals and Oils on Gut Health of European Sea Bass (*Dicentrarchus labrax*). Aquaculture.

[21] Rimoldi S., Gini E., Koch J.F.A., Iannini F., Brambilla F., Terova G. (2020). Erratum: Effects of Hydrolyzed Fish Protein and Autolyzed Yeast as Substitutes of Fishmeal in the Gilthead Sea Bream (*Sparus aurata*) Diet, on Fish Intestinal Microbiome. BMC Vet. Res..

[22] Moroni F., Naya-Català F., Piazzon M.C., Rimoldi S., Calduch-Giner J., Giardini A., Martínez I., Brambilla F., Pérez-Sánchez J., Terova G. (2021). The Effects of Nisin-Producing Lactococcus Lactis Strain Used as Probiotic on Gilthead Sea Bream (*Sparus aurata*) Growth, Gut Microbiota, and Transcriptional Response. Front. Mar. Sci..

[23] Piazzon M.C., Naya-Català F., Perera E., Palenzuela O., Sitjà-Bobadilla A., Pérez-Sánchez J. (2020). Genetic Selection for Growth Drives Differences in Intestinal Microbiota Composition and Parasite Disease Resistance in Gilthead Sea Bream. Microbiome.

[24] Naya-Català F., Piazzon M.C., Calduch-Giner J.A., Sitjà-Bobadilla A., Pérez-Sánchez J. (2022). Diet and Host Genetics Drive the Bacterial and Fungal Intestinal Metatranscriptome of Gilthead Sea Bream. Front. Microbiol..

[25] Firmino J.P., Vallejos-Vidal E., Balebona M.C., Ramayo-Caldas Y., Cerezo I.M., Salomón R., Tort L., Estevez A., Moriñigo M.Á., Reyes-López F.E. (2021). Diet, Immunity, and Microbiota Interactions: An Integrative Analysis of the Intestine Transcriptional Response and Microbiota Modulation in Gilthead Seabream (*Sparus aurata*) Fed an Essential Oils-Based Functional Diet. Front. Immunol..

[26] Piazzon M.C., Naya-Català F., Simó-Mirabet P., Picard-Sánchez A., Roig F.J., Calduch-Giner J.A., Sitjà-Bobadilla A., Pérez-Sánchez J. (2019). Sex, Age, and Bacteria: How the Intestinal Microbiota Is Modulated in a Protandrous Hermaphrodite Fish. Front. Microbiol..

[27] Infante-Villamil S., Huerlimann R., Jerry D.R. (2021). Microbiome Diversity and Dysbiosis in Aquaculture. Rev. Aquac..

[28] Pelusio N.F., Scicchitano D., Parma L., Dondi F., Brini E., D’Amico F., Candela M., Yúfera M., Gilannejad N., Moyano F.J. (2021). Interaction Between Dietary Lipid Level and Seasonal Temperature Changes in Gilthead Sea Bream Sparus Aurata: Effects on Growth, Fat Deposition, Plasma Biochemistry, Digestive Enzyme Activity, and Gut Bacterial Community. Front. Mar. Sci..

[29] Montero D., Serradell A., Gines R., Fontanillas R., Acosta F., Zamorano M.J., Fernández-Montero A., Pérez C., Afonso J.M., Torrecillas S. (2022). Nutritional innovations in superior European seabream (*Sparus aurata*) genotypes: Implications in fish performance. Proceedings of the Abstract Book of the XX International Symposium on Fish Nutrition and Feeding, Sorrento, Italy, June 2022.

[30] Hao W.-L., Lee Y.-K. (2004). Microflora of the gastrointestinal tract: A review. Methods Mol. Biol..

[31] Yang H.L., Sun Y.Z., Hu X., Ye J., Lu K.L., Hu L.H., Zhang J.-J. (2019). *Bacillus pumilus* SE5 originated PG and LTA tuned the intestinal TLRs/MyD88 signaling and microbiota in grouper (*Epinephelus coioides*). Fish Shellfish Immunol..

[32] Altschul S.F., Gish W., Miller W., Myers E.W., Lipman D.J. (1990). Basic Local Alignment Search Tool. J. Mol. Biol..

[33] Rognes T., Flouri T., Nichols B., Quince C., Mahé F. (2016). VSEARCH: A Versatile Open Source Tool for Metagenomics. PeerJ.

[34] Cole J.R., Wang Q., Fish J.A., Chai B., McGarrell D.M., Sun Y., Brown C.T., Porras-Alfaro A., Kuske C.R., Tiedje J.M. (2014). Ribosomal Database Project: Data and Tools for High Throughput rRNA Analysis. Nucleic Acids Res..

[35] Caicedo H.H., Hashimoto D.A., Caicedo J.C., Pentland A., Pisano G.P. (2020). Overcoming Barriers to Early Disease Intervention. Nat. Biotechnol..

[36] Bolger A.M., Lohse M., Usadel B. (2014). Trimmomatic: A Flexible Trimmer for Illumina Sequence Data. Bioinformatics.

[37] Dobin A., Davis C.A., Schlesinger F., Drenkow J., Zaleski C., Jha S., Batut P., Chaisson M., Gingeras T.R. (2013). STAR: Ultrafast Universal RNA-Seq Aligner. Bioinformatics.

[38] Pérez-Sánchez J., Naya-Català F., Soriano B., Piazzon M.C., Hafez A., Gabaldón T., Llorens C., Sitjà-Bobadilla A., Calduch-Giner J.A. (2019). Genome Sequencing and Transcriptome Analysis Reveal Recent Species-Specific Gene Duplications in the Plastic Gilthead Sea Bream (Sparus Aurata). Front. Mar. Sci..

[39] Liao Y., Smyth G.K., Shi W. (2019). The R Package Rsubread Is Easier, Faster, Cheaper and Better for Alignment and Quantification of RNA Sequencing Reads. Nucleic Acids Res..

[40] McMurdie P.J., Holmes S. (2013). Phyloseq: An R Package for Reproducible Interactive Analysis and Graphics of Microbiome Census Data. PLoS ONE.

[41] Wold S., Sjöström M., Eriksson L. (2001). PLS-Regression: A Basic Tool of Chemometrics. Chemom. Intell. Lab. Syst..

[42] Li H., Ma M.L., Luo S., Zhang R.M., Han P., Hu W. (2012). Metabolic Responses to Ethanol in Saccharomyces Cerevisiae Using a Gas Chromatography Tandem Mass Spectrometry-Based Metabolomics Approach. Int. J. Biochem. Cell Biol..

[43] Kieffer D.A., Piccolo B.D., Vaziri N.D., Liu S., Lau W.L., Khazaeli M., Nazertehrani S., Moore M.E., Marco M.L., Martin R.J. (2016). Resistant Starch Alters Gut Microbiome and Metabolomic Profiles Concurrent with Amelioration of Chronic Kidney Disease in Rats. Am. J. Physiol.-Ren. Physiol..

[44] Thévenot E.A., Roux A., Xu Y., Ezan E., Junot C. (2015). Analysis of the Human Adult Urinary Metabolome Variations with Age, Body Mass Index, and Gender by Implementing a Comprehensive Workflow for Univariate and OPLS Statistical Analyses. J. Proteome Res..

[45] Segata N., Izard J., Waldron L., Gevers D., Miropolsky L., Garrett W.S., Huttenhower C. (2011). Metagenomic Biomarker Discovery and Explanation. Genome Biol..

[46] Afgan E., Baker D., Batut B., Van Den Beek M., Bouvier D., Ech M., Chilton J., Clements D., Coraor N., Grüning B.A. (2018). The Galaxy Platform for Accessible, Reproducible and Collaborative Biomedical Analyses: 2018 Update. Nucleic Acids Res..

[47] Love M.I., Huber W., Anders S. (2014). Moderated Estimation of Fold Change and Dispersion for RNA-Seq Data with DESeq2. Genome Biol..

[48] Weiss S., Van Treuren W., Lozupone C., Faust K., Friedman J., Deng Y., Xia L.C., Xu Z.Z., Ursell L., Alm E.J. (2016). Correlation Detection Strategies in Microbial Data Sets Vary Widely in Sensitivity and Precision. ISME J..

[49] Smoot M.E., Ono K., Ruscheinski J., Wang P.L., Ideker T. (2011). Cytoscape 2.8: New Features for Data Integration and Network Visualization. Bioinformatics.

[50] Young M.D., Wakefield M.J., Smyth G.K., Oshlack A. (2010). Gene Ontology Analysis for RNA-Seq: Accounting for Selection Bias. Genome Biol..

[51] Klopfenstein D.V., Zhang L., Pedersen B.S., Ramírez F., Vesztrocy A.W., Naldi A., Mungall C.J., Yunes J.M., Botvinnik O., Weigel M. (2018). GOATOOLS: A Python Library for Gene Ontology Analyses. Sci. Rep..

[52] Väremo L., Nielsen J., Nookaew I. (2013). Enriching the Gene Set Analysis of Genome-Wide Data by Incorporating Directionality of Gene Expression and Combining Statistical Hypotheses and Methods. Nucleic Acids Res..

[53] Szklarczyk D., Gable A.L., Lyon D., Junge A., Wyder S., Huerta-Cepas J., Simonovic M., Doncheva N.T., Morris J.H., Bork P. (2019). STRING V11: Protein-Protein Association Networks with Increased Coverage, Supporting Functional Discovery in Genome-Wide Experimental Datasets. Nucleic Acids Res..

[54] Sea G., Sparus B., Nikouli E., Meziti A., Antonopoulou E., Mente E., Kormas K.A. (2019). Embryonic Stages and First Feeding in Farmed. Genes.

[55] Li X., Zhou L., Yu Y., Ni J., Xu W., Yan Q. (2017). Composition of Gut Microbiota in the Gibel Carp (*Carassius auratus* Gibelio) Varies with Host Development. Microb. Ecol..

[56] Lokesh J., Kiron V., Sipkema D., Fernandes J.M.O., Moum T. (2019). Succession of Embryonic and the Intestinal Bacterial Communities of Atlantic Salmon (*Salmo salar*) Reveals Stage-Specific Microbial Signatures. Microbiologyopen.

[57] Egerton S., Culloty S., Whooley J., Stanton C., Ross R.P. (2018). The Gut Microbiota of Marine Fish. Front. Microbiol..

[58] Anandan R., Dharumadurai D.M.G.P. (2016). An Introduction to Actinobacteria. Actinobacteria-Basics and Biotechnological Applications.

[59] Xie M., Xie Y., Li Y., Zhou W., Zhang Z., Yang Y., Olsen R.E., Ringø E., Ran C., Zhou Z. (2022). Stabilized Fermentation Product of *Cetobacterium somerae* Improves Gut and Liver Health and Antiviral Immunity of Zebrafish. Fish Shellfish Immunol..

[60] Sullam K.E., Essinger S.D., Lozupone C.A., Connor M.P.O. (2009). Environmental and Ecological Factors That Shape the Gut 2 Bacterial Communities of Fish: A Meta-Analysis-Supplementary. PubMed. Cent..

[61] Tarnecki A.M., Burgos F.A., Ray C.L., Arias C.R. (2017). Fish Intestinal Microbiome: Diversity and Symbiosis Unravelled by Metagenomics. J. Appl. Microbiol..

[62] Estruch G., Collado M.C., Peñaranda D.S., Tomás Vidal A., Jover Cerdá M., Pérez Martínez G., Martinez-Llorens S., Moreau C.S. (2015). Impact of Fishmeal Replacement in Diets for Gilthead Sea Bream (*Sparus aurata*) on the Gastrointestinal Microbiota Determined by Pyrosequencing the 16S RRNA Gene. PLoS ONE.

[63] Parma L., Candela M., Soverini M., Turroni S., Consolandi C., Brigidi P., Mandrioli L., Sirri R., Fontanillas R., Gatta P.P. (2016). Next-Generation Sequencing Characterization of the Gut Bacterial Community of Gilthead Sea Bream (*Sparus aurata*, L.) Fed Low Fishmeal Based Diets with Increasing Soybean Meal Levels. Anim. Feed Sci. Technol..

[64] Nikouli E., Meziti A., Antonopoulou E., Mente E., Kormas K.A. (2018). Gut Bacterial Communities in Geographically Distant Populations of Farmed Sea Bream (*Sparus aurata*) and Sea Bass (*Dicentrarchus labrax*). Microorganisms.

[65] Louca S., Jacques S.M.S., Pires A.P.F., Leal J.S., Srivastava D.S., Parfrey L.W., Farjalla V.F., Doebeli M. (2017). High Taxonomic Variability despite Stable Functional Structure across Microbial Communities. Nat. Ecol. Evol..

[66] Abdelhafiz Y., Fernandes J.M.O., Donati C., Pindo M., Kiron V. (2022). Intergenerational Transfer of Persistent Bacterial Communities in Female Nile Tilapia. Front. Microbiol..

[67] Roeselers G., Mittge E.K., Stephens W.Z., Parichy D.M., Cavanaugh C.M., Guillemin K., Rawls J.F. (2011). Evidence for a Core Gut Microbiota in the Zebrafish. ISME J..

[68] Dulski T., Kozłowski K., Ciesielski S. (2020). Habitat and Seasonality Shape the Structure of Tench (*Tinca tinca* L.) Gut Microbiome. Sci. Rep..

[69] Bereded N.K., Abebe G.B., Fanta S.W., Curto M., Waidbacher H., Meimberg H., Domig K.J. (2021). The Impact of Sampling Season and Catching Site (Wild and Aquaculture) on Gut Microbiota Composition and Diversity of Nile Tilapia (*Oreochromis niloticus*). Biology.

[70] Zhang Z., Li D., Refaey M.M., Xu W., Tang R., Li L. (2018). Host Age Affects the Development of Southern Catfish Gut Bacterial Community Divergent from That in the Food and Rearing Water. Front. Microbiol..

[71] Stephens W.Z., Burns A.R., Stagaman K., Wong S., Rawls J.F., Guillemin K., Bohannan B.J.M. (2016). The Composition of the Zebrafish Intestinal Microbial Community Varies across Development. ISME J..

[72] Small C.M., Currey M., Beck E.A., Bassham S., Cresko W.A. (2019). Highly Reproducible 16S Sequencing Facilitates Measurement of Host Genetic Influences on the Stickleback Gut Microbiome. mSystems.

[73] Steury R.A., Currey M.C., Cresko W.A., Bohannan B.J.M. (2019). Population Genetic Divergence and Environment Influence the Gut Microbiome in Oregon Threespine Stickleback. Genes.

[74] Blaufuss P.C., Bledsoe J.W., Gaylord T.G., Sealey W.M., Overturf K.E., Powell M.S. (2020). Selection on a Plant-Based Diet Reveals Changes in Oral Tolerance, Microbiota and Growth in Rainbow Trout (*Oncorhynchus mykiss*) When Fed a High Soy Diet. Aquaculture.

[75] Biasato I., Rimoldi S., Caimi C., Bellezza Oddon S., Chemello G., Prearo M., Saroglia M., Hardy R., Gasco L., Terova G. (2022). Efficacy of Utilization of All-Plant-Based and Commercial Low-Fishmeal Feeds in Two Divergently Selected Strains of Rainbow Trout (*Oncorhynchus mykiss*): Focus on Growth Performance, Whole-Body Proximate Composition, and Intestinal Microbiome. Front. Physiol..

[76] Koskinen R., TAli-Vehmas, Kämpfer P., Laurikkala M., Tsitko I., Kostyal E., Atroshi F., Salkinoja-Salonen M. (2000). Characterization of *Sphingomonas* Isolates from Finnish and Swedish Drinking Water Distribution Systems. J. Appl. Microbiol..

[77] Méndez-Pérez R., García-López R., Bautista-López J.S., Vázquez-Castellanos J., Alvarez-González C., Peña-Marín E., Baltierra-Trejo E., Adams-Schroeder R., Domínguez-Rodríguez V., Melgar-Valdés C. (2020). High-Throughput Sequencing of the 16S rRNA Gene to Analyze the Gut Microbiome in Juvenile and Adult Tropical Gar (*Atractosteus tropicus*). Lat. Am. J. Aquat. Res..

[78] Cui Y., Chun S.J., Ko S.R., Lee H.G., Srivastava A., Oh H.M., Ahn C.Y. (2017). *Reyranella aquatilis* Sp. Nov., an Alphaproteobacterium Isolated from a Eutrophic Lake. Int. J. Syst. Evol. Microbiol..

[79] Ikeda-Ohtsubo W., Brugman S., Warden C.H., Rebel J.M.J., Folkerts G., Pieterse C.M.J. (2018). How Can We Define “Optimal Microbiota?”: A Comparative Review of Structure and Functions of Microbiota of Animals, Fish, and Plants in Agriculture. Front. Nutr..

[80] Smith C.C.R., Snowberg L.K., Gregory Caporaso J., Knight R., Bolnick D.I. (2015). Dietary Input of Microbes and Host Genetic Variation Shape Among-Population Differences in Stickleback Gut Microbiota. ISME J..

[81] Benedito-Palos L., Ballester-Lozano G.F., Simó P., Karalazos V., Ortiz Á., Calduch-Giner J., Pérez-Sánchez J. (2016). Lasting Effects of Butyrate and Low FM/FO Diets on Growth Performance, Blood Haematology/Biochemistry and Molecular Growth-Related Markers in Gilthead Sea Bream (*Sparus aurata*). Aquaculture.

[82] Egerton S., Wan A., Murphy K., Collins F., Ahern G., Sugrue I., Busca K., Egan F., Muller N., Whooley J. (2020). Replacing Fishmeal with Plant Protein in Atlantic Salmon (*Salmo salar*) Diets by Supplementation with Fish Protein Hydrolysate. Sci. Rep..

[83] Perera E., Simó-Mirabet P., Shin H.S., Rosell-Moll E., Naya-Catalá F., de las Heras V., Martos-Sitcha J.A., Karalazos V., Armero E., Arizcun M. (2019). Selection for Growth Is Associated in Gilthead Sea Bream (*Sparus aurata*)with Diet Flexibility, Changes in Growth Patterns and Higher Intestine Plasticity. Aquaculture.

[84] Simó-Mirabet P., Felip A., Estensoro I., Martos-Sitcha J.A., de las Heras V., Calduch-Giner J., Puyalto M., Karalazos V., Sitjà-Bobadilla A., Pérez-Sánchez J. (2018). Impact of Low Fish Meal and Fish Oil Diets on the Performance, Sex Steroid Profile and Male-Female Sex Reversal of Gilthead Sea Bream (*Sparus aurata*) over a Three-Year Production Cycle. Aquaculture.

[85] Estensoro I., Ballester-Lozano G., Benedito-Palos L., Grammes F., Martos-Sitcha J.A., Mydland L.T., Calduch-Giner J.A., Fuentes J., Karalazos V., Ortiz Á. (2016). Dietary Butyrate Helps to Restore the Intestinal Status of a Marine Teleost (*Sparus aurata*) Fed Extreme Diets Low in Fish Meal and Fish Oil. PLoS ONE.

[86] Karlsen C., Tzimorotas D., Robertsen E.M., Kirste K.H., Bogevik A.S., Rud I. (2022). Feed microbiome: Confounding factor affecting fish gut microbiome studies. ISME Commun..

[87] Perez-Pascual D., Vendrell-Fernandez S., Audrain B., Bernal-Bayard J., Patiño-Navarrete R., Petit V., Rigaudeau D., Ghigo J.M. (2021). Gnotobiotic Rainbow Trout (*Oncorhynchus mykiss*) Model Reveals Endogenous Bacteria That Protect against *Flavobacterium columnare* Infection. PLoS Pathog..

[88] Ferrocino I., Rantsiou K., Cocolin L. (2022). Microbiome and -Omics Application in Food Industry. Int. J. Food Microbiol..

[89] Martin S.A.M., Dehler C.E., Król E. (2016). Transcriptomic Responses in the Fish Intestine. Dev. Comp. Immunol..

[90] Nichols R.G., Davenport E.R. (2021). The Relationship between the Gut Microbiome and Host Gene Expression: A Review. Hum. Genet..

[91] Kurek K., Piotrowska D.M., Wiesiołek P., Chabowski A., Zendzian-Piotrowska M. (2012). [Role of Sphingolipids in Digestive System]. Postepy Hig. Med. Dosw. (Online).

[92] Li Y., Nicholson R.J., Summers S.A. (2022). Ceramide Signaling in the Gut. Mol. Cell. Endocrinol..

[93] Gil-Solsona R., Calduch-Giner J.A., Nácher-Mestre J., Lacalle-Bergeron L., Sancho J.V., Hernández F., Pérez-Sánchez J. (2019). Contributions of MS Metabolomics to Gilthead Sea Bream (*Sparus aurata*) Nutrition. Serum Fingerprinting of Fish Fed Low Fish Meal and Fish Oil Diets. Aquaculture.

[94] Harayama T., Riezman H. (2018). Understanding the Diversity of Membrane Lipid Composition. Nat. Rev. Mol. Cell Biol..

[95] Hishikawa D., Shindou H., Kobayashi S., Nakanishi H., Taguchi R., Shimizu T. (2008). Discovery of a Lysophospholipid Acyltransferase Family Essential for Membrane Asymmetry and Diversity. Proc. Natl. Acad. Sci. USA.

[96] Wang B., Tontonoz P. (2019). Phospholipid Remodeling in Physiology and Disease. Annu. Rev. Physiol..

[97] Benedito-Palos L., Ballester-Lozano G., Pérez-Sánchez J. (2014). Wide-Gene Expression Analysis of Lipid-Relevant Genes in Nutritionally Challenged Gilthead Sea Bream (*Sparus aurata*). Gene.

[98] Okada T., Fukuda S., Hase K., Nishiumi S., Izumi Y., Yoshida M., Hagiwara T., Kawashima R., Yamazaki M., Oshio T. (2013). Microbiota-Derived Lactate Accelerates Colon Epithelial Cell Turnover in Starvation-Refed Mice. Nat. Commun..

[99] Janssen P., Vanden Berghe P., Verschueren S., Lehmann A., Depoortere I., Tack J. (2011). Review Article: The Role of Gastric Motility in the Control of Food Intake. Aliment. Pharmacol. Ther..

[100] Wang M., Wang L., Tan X., Wang L., Xiong X., Wang Y., Wang Q., Yang H., Yin Y. (2022). The Developmental Changes in Intestinal Epithelial Cell Proliferation, Differentiation, and Shedding in Weaning Piglets. Anim. Nutr..

[101] Patel K.S., Thavamani A. (2022). Physiology, Peristalsis. StatPearls [Internet].

[102] Yuan J., Gao Y., Sun L., Jin S., Zhang X., Liu C., Li F., Xiang J. (2019). Wnt Signaling Pathway Linked to Intestinal Regeneration via Evolutionary Patterns and Gene Expression in the Sea Cucumber *Apostichopus japonicus*. Front. Genet..

[103] Perochon J., Carroll L.R., Cordero J.B. (2018). Wnt Signalling in Intestinal Stem Cells: Lessons from Mice and Flies. Genes.

[104] Huizinga J.D., Chen J.H., Zhu Y.F., Pawelka A., McGinn R.J., Bardakjian B.L., Parsons S.P., Kunze W.A., Wu R.Y., Bercik P. (2014). The Origin of Segmentation Motor Activity in the Intestine. Nat. Commun..

[105] Kataoka K., Rikitake Y., Ayabe Y. (2015). Expression Pattern of Dkk-3, a Secreted Wnt Pathway Inhibitor, in Mouse Intestinal Tissue and Three-Dimensional Cultured Caco-2 Spheroids. J. Stem Cells Regen. Med..

[106] Prühs R., Beermann A., Schröder R. (2017). The Roles of the Wnt-Antagonists Axin and Lrp4 during Embryogenesis of the Red Flour Beetle Tribolium Castaneum. J. Dev. Biol..

[107] Berthelot C.C., Kamita S.G., Sacchi R., Yang J., Nording M.L., Georgi K., Karbowski C.H., German J.B., Weiss R.H., Hogg R.J. (2015). Changes in PTGS1 and ALOX12 Gene Expression in Peripheral Blood Mononuclear Cells Are Associated with Changes in Arachidonic Acid, Oxylipins, and Oxylipin/Fatty Acid Ratios in Response to Omega-3 Fatty Acid Supplementation. PLoS ONE.

[108] Wang Q., Lin Y., Sheng X., Xu J., Hou X., Li Y., Zhang H., Guo H., Yu Z., Ren F. (2020). Arachidonic Acid Promotes Intestinal Regeneration by Activating WNT Signaling. Stem Cell Reports.

[109] Gela A., Jovic S., Nordin S.L., Egesten A. (2014). Midkine in Host Defence. Br. J. Pharmacol..

[110] Nilojan J., Bathige S.D.N.K., Kugapreethan R., Thulasitha W.S., Nam B.H., Lee J. (2017). Molecular, Transcriptional and Functional Insights into Duplicated Goose-Type Lysozymes from *Sebastes schlegelii* and Their Potential Immunological Role. Fish Shellfish Immunol..

[111] Zupok A., Iobbi-Nivol C., Méjean V., Leimkühler S. (2019). The Regulation of Moco Biosynthesis and Molybdoenzyme Gene Expression by Molybdenum and Iron in Bacteria. Metallomics.

[112] Lin T.L., Shu C.C., Chen Y.M., Lu J.J., Wu T.S., Lai W.F., Tzeng C.M., Lai H.C., Lu C.C. (2020). Like Cures Like: Pharmacological Activity of Anti-Inflammatory Lipopolysaccharides From Gut Microbiome. Front. Pharmacol..

[113] Komori T., Saito K., Sawa N., Shibasaki Y., Kohchi C., Soma G.I., Inagawa H. (2015). Innate Immunity Activated by Oral Administration of Lpsp Is Phylogenetically Preserved and Developed in Broiler Chickens. Anticancer Res..

[114] Fink M.P. (2014). Animal Models of Sepsis. Virulence.

[115] Philip A.M., Wang Y., Mauro A., El-Rass S., Marshall J.C., Lee W.L., Slutsky A.S., dos Santos C.C., Wen X.Y. (2017). Development of a Zebrafish Sepsis Model for High-Throughput Drug Discovery. Mol. Med..

[116] Kelley N., Jeltema D., Duan Y., He Y. (2019). The NLRP3 Inflammasome: An Overview of Mechanisms of Activation and Regulation. Int. J. Mol. Sci..

[117] Feng Y., Wang Y., Wang P., Huang Y., Wang F. (2018). Short-Chain Fatty Acids Manifest Stimulative and Protective Effects on Intestinal Barrier Function Through the Inhibition of NLRP3 Inflammasome and Autophagy. Cell. Physiol. Biochem..

[118] Man S.M. (2018). Inflammasomes in the Gastrointestinal Tract: Infection, Cancer and Gut Microbiota Homeostasis. Nat. Rev. Gastroenterol. Hepatol..

[119] Xue J., Ajuwon K.M., Fang R. (2020). Mechanistic Insight into the Gut Microbiome and Its Interaction with Host Immunity and Inflammation. Anim. Nutr..

[120] Cheng D., Xu J.H., Li J.Y., Wang S.Y., Wu T.F., Chen Q.K., Yu T. (2018). Butyrate Ameliorated-NLRC3 Protects the Intestinal Barrier in a GPR43-Dependent Manner. Exp. Cell Res..

[121] Yan S., Yang B., Zhao J., Zhao J., Stanton C., Ross R.P., Zhang H., Chen W. (2019). A Ropy Exopolysaccharide Producing Strain: *Bifidobacterium longum* Subsp. *longum* YS108R Alleviates DSS-Induced Colitis by Maintenance of the Mucosal Barrier and Gut Microbiota Modulation. Food Funct..

[122] Giri S.S., Ryu E.C., Sukumaran V., Park S.C. (2019). Antioxidant, Antibacterial, and Anti-Adhesive Activities of Biosurfactants Isolated from *Bacillus* Strains. Microb. Pathog..

[123] Simó-Mirabet P., Piazzon M.C., Calduch-Giner J.A., Ortiz Á., Puyalto M., Sitjà-Bobadilla A., Pérez-Sánchez J. (2017). Sodium Salt Medium-Chain Fatty Acids and *Bacillus*-Based Probiotic Strategies to Improve Growth and Intestinal Health of Gilthead Sea Bream (*Sparus aurata*). PeerJ.

[124] Mingmongkolchai S., Panbangred W. (2018). Bacillus Probiotics: An Alternative to Antibiotics for Livestock Production. J. Appl. Microbiol..

[125] Alfano M., Canducci F., Nebuloni M., Clementi M., Montorsi F., Salonia A. (2016). The Interplay of Extracellular Matrix and Microbiome in Urothelial Bladder Cancer. Nat. Rev. Urol..

[126] Ma X., Zhu F. (2021). The Role of Myosin-9 in *Scylla paramamosain* against *Vibrio alginolyticus* and White Spot Syndrome Virus Infection. Aquaculture.

[127] Zarȩba T.W., Pascu C., Hryniewicz W., Wadström T. (1997). Binding of Extracellular Matrix Proteins by Enterococci. Curr. Microbiol..

