# Supplementary material for: Genetics and Nutrition Drive the Gut Microbiota Succession and Host-Transcriptome Interactions through the Gilthead Sea Bream (Sparus aurata) Production Cycle
Source: Biology (Basel). 2022 Nov 30;11(12):1744. doi: 10.3390/biology11121744 (PMC9774573; doi:10.3390/biology11121744)
Supplement: Supplementary file 1 [file biology-11-01744-s001.zip › Supplementary Table S1.pdf]

Supplementary Table S1. Table showing the detailed 16S amplicon-based sequencing data obtained in this study, as well as the number of observed OTUs and the species richness estimator (Chao1), obtained for each sample.

Time	Sample	Raw reads	Assigned reads	Observed OTUs	Chao1
t_0	GS-1	189745	87976	93	444.00
t_0	GS-2	197092	120415	159	200.35
t_0	GS-3	171615	148220	130	283.11
t_0	GS-4	197468	149398	85	320.20
t_0	GS-5	201842	87835	126	1072.00
t_0	GS-6	211897	123394	97	135.75
t_0	GS-7	176647	84559	93	621.00
t_0	REF-1	206016	129085	177	293.37
t_0	REF-2	169041	149257	179	685.67
t_0	REF-3	177883	164355	158	668.00
t_0	REF-4	203385	140417	326	389.98
t_0	REF-5	205011	116160	334	526.60
t_0	REF-6	210377	122552	154	239.56
t_0	REF-7	198386	98152	219	292.75
t_0	REF-8	166924	63448	146	146.00
t_0	REF-9	163096	147138	121	170.79
t_0	REF-10	137690	111992	138	257.25
t_0	REF-11	136923	128432	176	281.12
t_1	GS-CTRL-1	151869	84385	106	571.00
t_1	GS-CTRL-2	221285	113975	125	189.50
t_1	GS-CTRL-3	234025	143782	140	186.87
t_1	GS-CTRL-4	344520	260368	88	103.00
t_1	GS-CTRL-5	237054	158885	135	262.50
t_1	GS-CTRL-6	202678	131122	91	134.15
t_1	GS-CTRL-7	226177	147802	111	134.80
t_1	GS-CTRL-8	225219	158125	155	277.77
t_1	GS-CTRL-9	173038	103360	87	129.00
t_1	GS-FUTURE-1	232407	122444	110	151.35
t_1	GS-FUTURE-2	305488	218239	130	354.00
t_1	GS-FUTURE-3	304096	116725	164	239.25
t_1	GS-FUTURE-4	227412	117105	99	126.00
t_1	GS-FUTURE-5	230786	127201	112	164.00
t_1	GS-FUTURE-6	221545	101611	115	283.00
t_1	GS-FUTURE-7	213915	97236	100	174.38
t_1	GS-FUTURE-8	241840	120101	118	184.00
t_1	GS-FUTURE-9	271655	180356	106	256.27
t_1	REF-CTRL-1	238813	139303	116	157.05

t_1	REF-CTRL-2	264191	103815	107	165.58
t_1	REF-CTRL-3	207281	148539	88	146.67
t_1	REF-CTRL-4	229544	189102	114	213.00
t_1	REF-CTRL-5	270132	134059	119	141.88
t_1	REF-CTRL-6	268118	121949	146	214.06
t_1	REF-CTRL-7	283067	142258	110	162.11
t_1	REF-CTRL-8	229491	177570	118	356.50
t_1	REF-CTRL-9	271959	116915	135	226.07
t_1	REF-FUTURE-1	272537	146570	120	138.50
t_1	REF-FUTURE-2	281251	156920	114	224.00
t_1	REF-FUTURE-3	242337	139390	117	162.32
t_1	REF-FUTURE-4	224019	139532	123	145.55
t_1	REF-FUTURE-5	290759	213803	92	239.00
t_1	REF-FUTURE-6	209210	127144	98	150.50
t_1	REF-FUTURE-7	208318	139450	84	95.00
t_1	REF-FUTURE-8	249769	131585	149	188.06
t_1	REF-FUTURE-9	229040	140759	107	154.14
t_2	GS-CTRL-1	143845	98086	67	154.75
t_2	GS-CTRL-2	88606	88570	98	839.00
t_2	GS-CTRL-3	106961	63569	100	100.00
t_2	GS-CTRL-4	123278	90829	100	272.50
t_2	GS-CTRL-5	82807	81900	109	775.00
t_2	GS-CTRL-6	85141	48827	45	45.00
t_2	GS-CTRL-7	96884	93364	68	69.73
t_2	GS-CTRL-8	123249	68836	34	34.00
t_2	GS-CTRL-9	142899	65075	56	56.00
t_2	GS-CTRL-10	108190	96318	65	104.43
t_2	GS-FUTURE-1	113477	75032	100	841.00
t_2	GS-FUTURE-2	108135	48030	86	86.00
t_2	GS-FUTURE-3	108930	68407	240	240.00
t_2	GS-FUTURE-4	133209	124580	60	118.00
t_2	GS-FUTURE-5	100354	64781	57	57.00
t_2	GS-FUTURE-6	103772	92918	46	131.50
t_2	GS-FUTURE-7	189898	116456	67	109.86
t_2	GS-FUTURE-8	117702	110077	61	62.17
t_2	GS-FUTURE-9	95071	60953	68	68.00
t_2	GS-FUTURE-10	66372	63791	64	64.00
t_2	REF-CTRL-1	82252	41917	51	51.00
t_2	REF-CTRL-2	183541	70185	109	109.00
t_2	REF-CTRL-3	122751	46291	110	110.00
t_2	REF-CTRL-4	245219	42352	35	35.00
t_2	REF-CTRL-5	113616	51493	44	44.00

t_2	REF-CTRL-6	144479	56421	69	69.00
t_2	REF-CTRL-7	86433	54025	75	75.00
t_2	REF-CTRL-8	117657	115040	71	72.24
t_2	REF-CTRL-9	67674	45138	64	64.00
t_2	REF-CTRL-10	179915	73368	73	73.00
t_2	REF-FUTURE-1	261728	49036	70	70.00
t_2	REF-FUTURE-2	97995	91842	79	219.60
t_2	REF-FUTURE-3	98003	91231	119	171.93
t_2	REF-FUTURE-4	78106	37566	58	58.00
t_2	REF-FUTURE-5	159954	95713	86	141.11
t_2	REF-FUTURE-6	282128	46723	55	55.00
t_2	REF-FUTURE-7	265983	72620	45	45.00
t_2	REF-FUTURE-8	115114	83535	39	39.00
t_2	REF-FUTURE-9	65347	61902	41	41.00
t_2	REF-FUTURE-10	91081	60342	54	54.00
