# Supplementary material for: Genetics and Nutrition Drive the Gut Microbiota Succession and Host-Transcriptome Interactions through the Gilthead Sea Bream (Sparus aurata) Production Cycle
Source: Biology (Basel). 2022 Nov 30;11(12):1744. doi: 10.3390/biology11121744 (PMC9774573; doi:10.3390/biology11121744)
Supplement: Supplementary file 1 [file biology-11-01744-s001.zip › Supplementary Table S4.pdf]

Supplementary Table S4. Pathway analysis from predicted metagenomes. Values represent the log2 fold change of the comparisons between GS and REF fish in the fish fed CTRL diet at t_0, t_1, and t_2.

**Pathway**	**t_0**	**t_1**	**t_2**
Xylene degradation	0.57		
Citrate cycle (TCA cycle)	0.44		
Carbohydrate metabolism	0.41		
Selenocompound metabolism	0.41		
C5-Branched dibasic acid metabolism	0.38	0.32	
RNA polymerase	0.37		
Aminoacyl-tRNA biosynthesis	0.36		
Valine leucine and isoleucine biosynthesis	0.33	0.19	
Cytoskeleton proteins	0.32		
Cysteine and methionine metabolism	0.27		
Sulfur metabolism	0.27		
Ribosome	0.22		
Peptidases	0.16		
One carbon pool by folate	0.12		
Non-homologous end-joining	-1.44		
Basal transcription factors	-5.88	-5.71	
Betalain biosynthesis	-6.74		
Indole alkaloid biosynthesis	-8.66		
Caffeine metabolism	-12.56		
Clavulanic acid biosynthesis	-21.99	-8.23	
Vasopressin-regulated water reabsorption	-30.00		
Shigellosis		11.07	
Metabolism of cofactors and vitamins		0.20	
Pantothenate and CoA biosynthesis		0.18	
Galactose metabolism		-0.47	
Primary bile acid biosynthesis		-1.26	
Steroid biosynthesis		-2.16	
Other glycan degradation		-2.37	
Glycosaminoglycan degradation		-3.44	
RIG-I-like receptor signaling pathway		-4.49	
Glycosphingolipid biosynthesis – lacto and neolacto series		-7.68	
Cytochrome P450			26.15
Hematopoietic cell lineage			26.15
