# Supplementary material for: Genetics and Nutrition Drive the Gut Microbiota Succession and Host-Transcriptome Interactions through the Gilthead Sea Bream (Sparus aurata) Production Cycle
Source: Biology (Basel). 2022 Nov 30;11(12):1744. doi: 10.3390/biology11121744 (PMC9774573; doi:10.3390/biology11121744)
Supplement: Supplementary file 1 [file biology-11-01744-s001.zip › Supplementary Table S5.pdf]

Supplementary Table S5. Sequencing and mapping metrics of the 36 samples included in the RNA-seq analysis.

Time	Sample	Raw reads	Preprocessed reads	Mapped reads	% mapping	% unique mapping
t_1	GS-CTRL-1	44384026	43328328	40868961	94.32	74.54
t_1	GS-CTRL-2	38917508	37817519	35547031	94.00	74.40
t_1	GS-CTRL-3	48137311	46859330	44427091	94.81	75.76
t_1	GS-CTRL-4	32969060	31884946	29936409	93.89	73.22
t_1	GS-CTRL-5	25714516	24912438	23525072	94.43	74.44
t_1	GS-CTRL-6	34243840	33138866	31097834	93.84	73.78
t_1	GS-CTRL-7	27167406	26413962	24832692	94.01	74.74
t_1	GS-CTRL-8	33277032	32240388	30287722	93.94	74.41
t_1	GS-CTRL-9	52097159	50646552	47492767	93.77	73.39
t_1	GS-FUTURE-1	18221552	17679966	16637016	94.10	74.91
t_1	GS-FUTURE-2	28777882	28050106	26010811	92.73	70.80
t_1	GS-FUTURE-3	28008516	27145185	25315981	93.26	71.30
t_1	GS-FUTURE-4	19062050	18410554	17232664	93.60	72.78
t_1	GS-FUTURE-5	30761362	29731529	27785645	93.46	73.78
t_1	GS-FUTURE-6	24684974	23954086	22236222	92.83	69.15
t_1	GS-FUTURE-7	25228022	24503474	22893785	93.43	72.00
t_1	GS-FUTURE-8	23072981	22357841	20533472	91.84	68.84
t_1	GS-FUTURE-9	19169954	18589031	17200210	92.53	70.21
t_1	REF-CTRL-1	29744974	28856602	27077432	93.83	73.02
t_1	REF-CTRL-2	33116289	32155826	30095798	93.59	72.94
t_1	REF-CTRL-3	37355105	36284421	34052428	93.85	72.73
t_1	REF-CTRL-4	20466088	19853441	18445706	92.91	70.04
t_1	REF-CTRL-5	18292194	17715126	16450054	92.86	70.82
t_1	REF-CTRL-6	22629336	21897685	20343706	92.90	71.51
t_1	REF-CTRL-7	23966902	23247015	21701122	93.35	72.01
t_1	REF-CTRL-8	25718415	24939927	23029905	92.34	70.49
t_1	REF-CTRL-9	21568822	20841943	19410420	93.13	71.50
t_1	REF-FUTURE-1	34720535	33749501	31274136	92.67	69.36
t_1	REF-FUTURE-2	30260267	29329700	27347647	93.24	72.06
t_1	REF-FUTURE-3	20094856	19429979	18041387	92.85	71.36
t_1	REF-FUTURE-4	38994107	37869344	34967434	92.34	70.33
t_1	REF-FUTURE-5	23434179	22698352	21161910	93.23	71.15
t_1	REF-FUTURE-6	19542795	18909857	17252307	91.23	67.32
t_1	REF-FUTURE-7	24158701	23476852	21515827	91.65	67.87
t_1	REF-FUTURE-8	36074803	35052958	32401876	92.44	69.87
t_1	REF-FUTURE-9	35776635	34730594	31984560	92.09	69.63
