# Supplementary material for: Genetics and Nutrition Drive the Gut Microbiota Succession and Host-Transcriptome Interactions through the Gilthead Sea Bream (Sparus aurata) Production Cycle
Source: Biology (Basel). 2022 Nov 30;11(12):1744. doi: 10.3390/biology11121744 (PMC9774573; doi:10.3390/biology11121744)
Supplement: Supplementary file 1 [file biology-11-01744-s001.zip › Supplementary Table S7.pdf]

Supplementary Table S7. Comparison between RNA-seq results and real-time PCR validation.

Gene Name	Symbol	GenBank accession	Fold change (Gs-FUTURE/GS-CTRL)	
			**RNA-seq**	**Real-time PCR**
Acyl-CoA desaturase	*scd1a*	JQ277703	-3.06±0.41	-4.14±0.27
Cathepsin S	*ctss*	KM522788	-2.88±0.31	-1.64±0.14
Fatty acid-binding protein, heart	*h-fabp*	JQ308834	1.24±0.18	1.36±0.09
Hepcidin	*hamp*	AM749960	1.99±0.34	1.85±0.16
Lysophospholipid acyltransferase 3	*lpcat3*	JQ277706	-1.13±0.14	-1.26±0.12
Vimentin	*des*	KF857332	2.88±0.42	3.35±0.22
