# Supplementary figures and images for: Genetics and Nutrition Drive the Gut Microbiota Succession and Host-Transcriptome Interactions through the Gilthead Sea Bream (Sparus aurata) Production Cycle
Source: Biology (Basel). 2022 Nov 30;11(12):1744. doi: 10.3390/biology11121744 (PMC9774573; doi:10.3390/biology11121744)

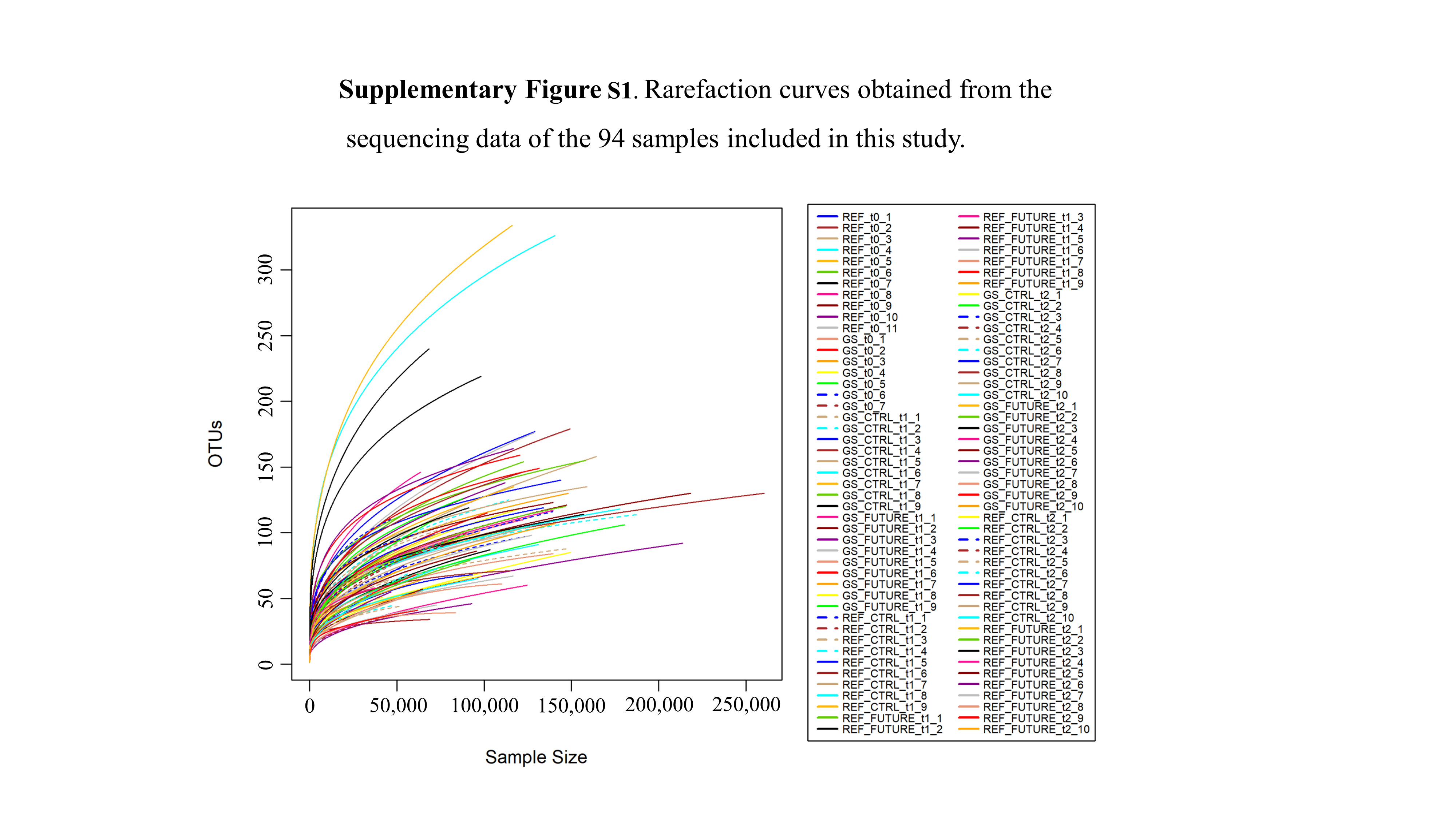

Supplement: Supplementary file 1 [file biology-11-01744-s001.zip › Supplementary Figure S1.jpg]

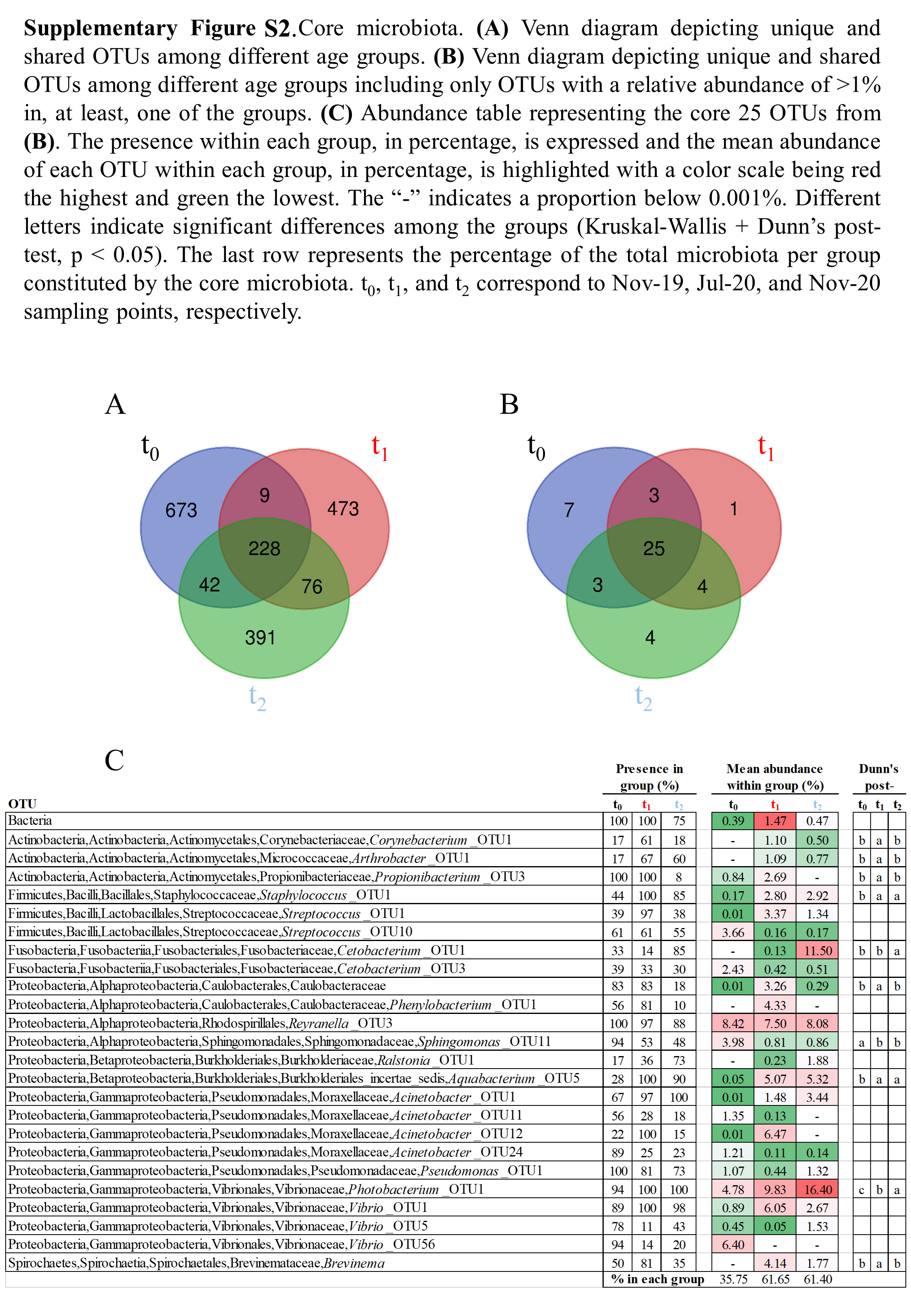

Supplement: Supplementary file 1 [file biology-11-01744-s001.zip › Supplementary Figure S2.jpg]

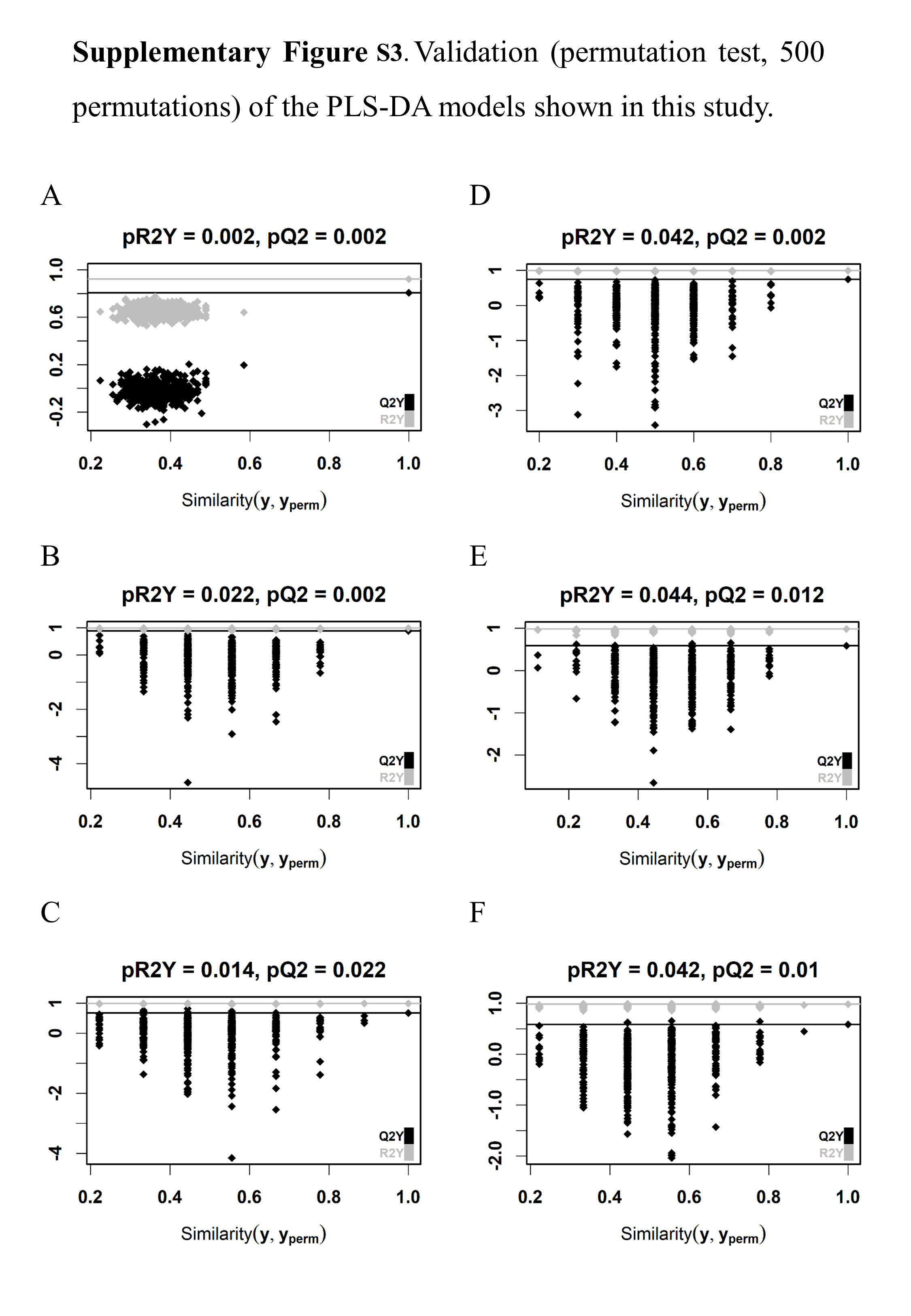

Supplement: Supplementary file 1 [file biology-11-01744-s001.zip › Supplementary Figure S3.jpg]

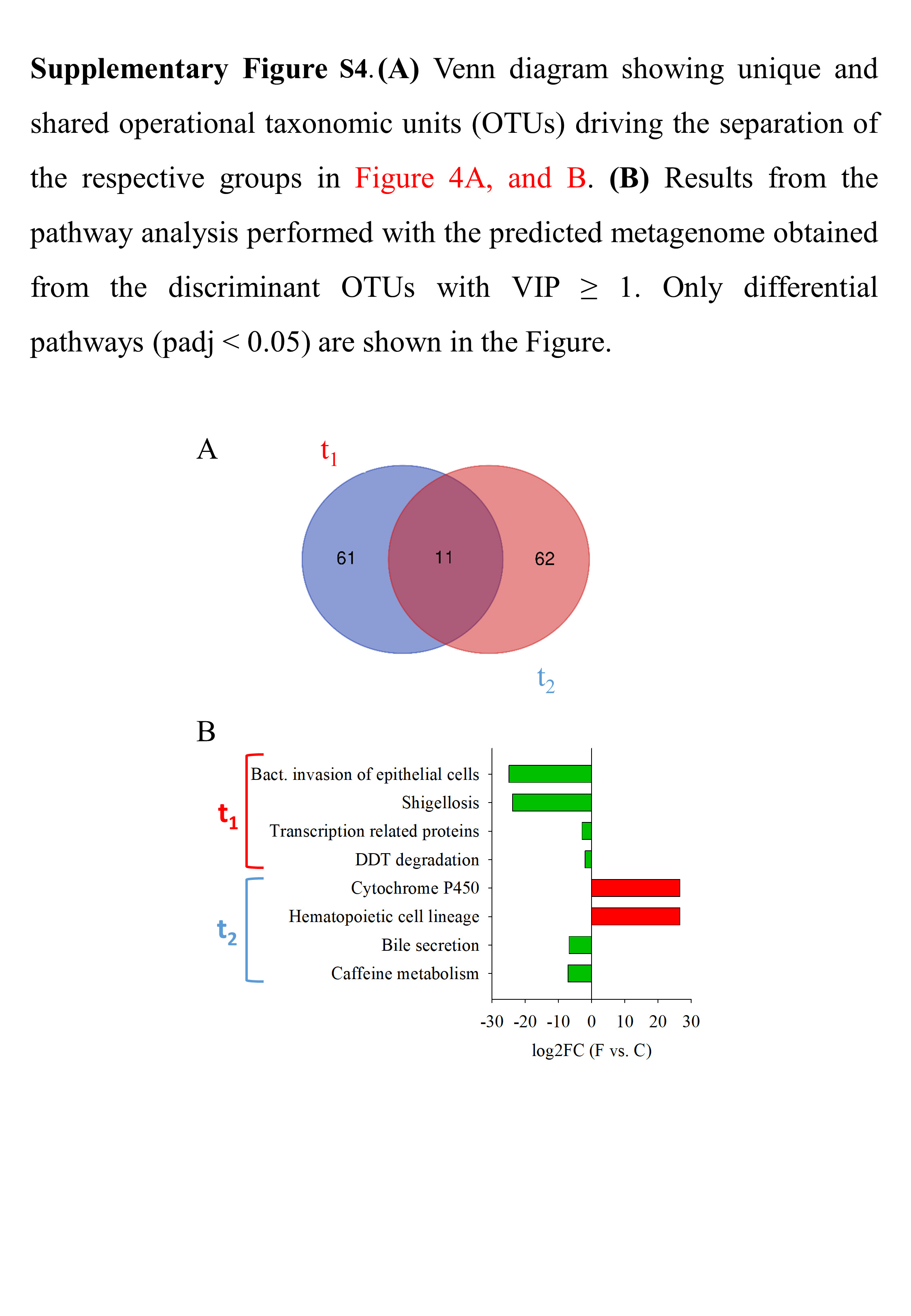

Supplement: Supplementary file 1 [file biology-11-01744-s001.zip › Supplementary Figure S4.jpg]
